# Degradable STING nanomodulators orchestrate the innate-to-adaptive immune response for NIR-II photothermal-immunotherapy via a cancer-immunity cycle

**DOI:** 10.1016/j.mtbio.2026.103028

**Published:** 2026-03-14

**Authors:** Qiaolin Wei, Zirui Zhu, Yue Li, Siying Sun, Ge Gao, Yinghong Wan, Yi Hao, Jiaying Lei, Jiahao Xu, Quan Hu, Wei Zheng, Yong Guo, Jia-Wei Shen

**Affiliations:** a Zhejiang Provincial Key Laboratory of Anti-Cancer Chinese Medicines and Natural Medicines, School of Pharmacy, Hangzhou Normal University, Zhejiang, 311121, China; b Engineering Laboratory of Development and Application of Traditional Chinese Medicines, Collaborative Innovation Center of Traditional Chinese Medicines of Zhejiang Province, Hangzhou Normal University, Hangzhou, Zhejiang, 311121, China; c College of Life and Environmental Sciences, Hangzhou Normal University, Hangzhou, Zhejiang, 311121, China

**Keywords:** cGAS-STING pathway, STING nanomodulators, Cancer-immunity cycle, Innate-to-adaptive immune response, NIR-II photothermal immunotherapy

## Abstract

Activating the cyclic GMP-AMP synthase-stimulator of interferon genes (cGAS-STING) pathway has become a valuable approach for enhancing the efficacy of immunotherapeutic treatments. Here, a degradable Au-Zn bimetallic STING nanomodulator (designated as GZn NPs) was constructed to coordinate the second near-infrared window (NIR-II) photothermal therapy and immunotherapy. Under acidic tumor microenvironment conditions, plus with NIR-II laser irradiation, GZn NPs undergo responsive degradation, releasing Zn^2+^ ions that directly enhance cGAS enzymatic activity. Concurrently, Zn^2+^ overload triggers excessive reactive oxygen species (ROS) generation, which, together with the photothermal effect of gold nanostars, produces a synergistic ROS burst. The resulting oxidative stress intensively damages mitochondria and the nucleus, promoting the accumulation of cytosolic dsDNA, thereby synergistically with Zn^2+^ activating the cGAS-STING signals. NIR-II photothermal therapy and ROS-mediated oxidative stress induce immunogenic cell death (ICD), which, combined with cGAS-STING pathway activation, promotes dendritic cells (DCs) maturation and T cell infiltration for primary tumor regression. This process also establishes long-term anti-tumor immunity to inhibit tumor recurrence, orchestrating the cycle from innate to adaptive immunity. Collectively, this study presents a promising STING nanomodulator that demonstrates the potential of NIR-II photothermal-immunotherapy in cancer treatment.

## Highlights

• GZn NPs represent a dual-responsive Au-Zn bimetallic STING nanomodulator for controlled Zn^2+^ release.
• GZn NPs induce ROS burst via Zn^2+^ homeostasis disruption and NIR-II photothermal therapy, causing severe oxidative stress.
• GZn NPs activate the cGAS-STING pathway through a dual role: Zn^2+^ potentiates cGAS enzymatic activity while synergizing with oxidative stress-induced dsDNA.
• GZn NPs activate the cGAS-STING pathway and immunogenic cell death, promoting T cell infiltration and immune memory.
• GZn NPs orchestrate the innate-to-adaptive immune response, establish a cancer-immunity cycle.

## Graphical abstract

This work presents a degradable Au-Zn bimetallic STING nanomodulator (GZn NPs). The construct degrades under acidic tumor microenvironment and NIR-II laser irradiation, causing Zn^2+^ release and a synergistic ROS burst. This dual outcome activates the cGAS-STING pathway and induces immunogenic cell death, which initiates an amplifying cancer-immunity cycle and ultimately achieves robust antitumor immunity and long-term immune memory.

**Figure:**
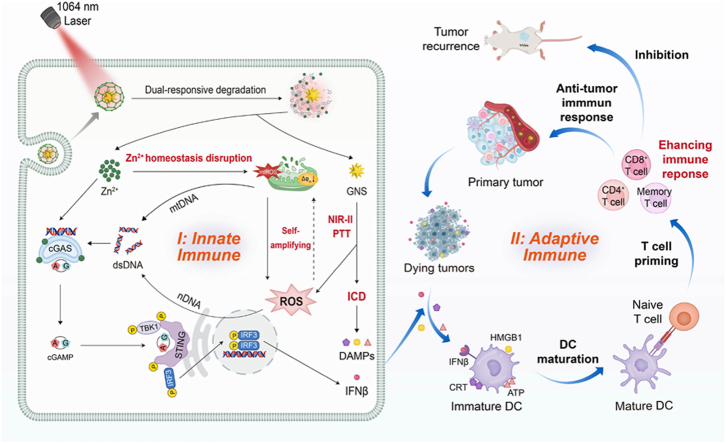

## 1 Introduction

Immunotherapy harnesses the patient's own immune system to recognize and eliminate tumor cells, offering distinct advantages such as high specificity, broad applicability, and sustained anti-tumor effects [[1], [2], [3]]. However, its effectiveness is often limited by inadequate immune cell infiltration due to the immunosuppressive tumor microenvironment (TME), resulting in a generally low clinical response rate [4]. Therefore, reprogramming the immunosuppressive TME to elicit a potent antitumor immune response has become a major focus of current research [[5], [6], [7], [8], [9], [10]]. Among them, activation of the cyclic GMP-AMP synthase (cGAS)-stimulator of interferon genes (STING) signaling pathway, a key innate immune signaling route, has emerged as a promising strategy to enhance tumor immunotherapy [[11], [12], [13], [14], [15], [16], [17]]. In tumor cells, cGAS can sense intracellular damaged DNA and activate STING, leading to the upregulation of type I interferons (IFN-I) and pro-inflammatory cytokines, thereby inhibiting tumor progression [18,19]. Additionally, tumor-derived DNA can also activate the cGAS-STING pathway in antigen-presenting cells (APCs), ultimately promoting and activating CD8^+^ T cells to initiate a robust adaptive immune response and improve the outcome of antitumor immunotherapy [20,21].

Immunogenic cell death (ICD) is a promising strategy for reprogramming the tumor immune microenvironment. During ICD, dying tumor cells release tumor-associated antigens (TAAs) and damage-associated molecular patterns (DAMPs) like calreticulin (CRT), high mobility group box 1 (HMGB1), and adenosine triphosphate (ATP). These signals facilitate dendritic cell (DC) maturation and the initiation of T cell-mediated immune responses, thereby activating adaptive immunity and enhancing tumor immunogenicity [[22], [23], [24]]. Photothermal therapy (PTT) not only directly eliminates tumor cells through high temperatures but also can induce ICD and stimulate a systemic antitumor immune response [[25], [26], [27], [28], [29]]. However, the ICD induced by single-modality PTT is often weak due to the impaired redox microenvironment and is insufficient to trigger a robust systemic antitumor immune response [30]. Noble metal nanomaterials, widely used as photothermal agents, can enhance energy conversion via the localized surface plasmon resonance (LSPR) effect to promote ROS generation, thereby amplifying oxidative stress and potentiating the immune effects of PTT [[31], [32], [33], [34], [35], [36], [37]]. This provides a new synergistic combination point for PTT and immunotherapy. Furthermore, the second near-infrared window (NIR-II) photothermal therapy, with deeper tissue penetration (around 3-5 cm), provides an ideal platform for developing more efficient PTT-immunotherapy [23,[38], [39], [40], [41]]. Notably, PTT itself can damage mitochondria and the nucleus, leading to leakage of mitochondrial DNA (mtDNA) and nuclear DNA (nDNA) into the cytoplasm. These double-stranded DNA (dsDNA) molecules are then sensed by cGAS, thus activating the cGAS-STING pathway [[42], [43], [44]]. In summary, by integrating photothermal effects with ROS-mediated oxidative stress, PTT can effectively enhance immunogenicity and augment antitumor immune responses.

The role of metal ions in immune modulation should not be overlooked [[45], [46], [47]]. In particular, zinc ion (Zn^2+^) homeostasis has been recognized to play a crucial role in the development, invasion, and metastasis of various tumors [48,49]. Zn^2+^ is a key regulator of the cGAS-STING signaling pathway, activating it through a dual mechanism. Firstly, Zn^2+^ directly promotes phase separation between cGAS and dsDNA, thereby enhancing cGAS enzymatic activity and triggering downstream STING signaling [50,51]. Secondly, disruption of Zn^2+^ homeostasis can impair mitochondrial electron transport chain (ETC) complexes, compromise mitochondrial function, and stimulate excessive production of mitochondrial superoxide anion (⋅O_2_^−^) [45,46,[52], [53], [54], [55], [56]]. This oxidative stress subsequently leads to cytosolic leakage of damaged dsDNA, which serves as an upstream signal that further activates the cGAS-STING pathway [57,58]. Consequently, dysregulation of Zn^2+^ homeostasis not only inhibits tumor progression but also offers a therapeutic opportunity via STING pathway activation. Collectively, leveraging the aforementioned mechanisms, metalloimmunotherapy represents a promising breakthrough strategy for remodeling the tumor immune microenvironment and enhancing antitumor immunotherapy through synergistic cGAS-STING pathway activation.

In this work, we developed dual-responsive Au-Zn bimetallic STING nanomodulators (designated as GZn NPs) to achieve tumor-specific activation of the cGAS-STING pathway and evaluated their potential in NIR-II photothermal-immunotherapy. The system consists of gold nanostars (GNS) as an NIR-II photothermal core coated with a zeolitic imidazolate framework-8 (ZIF-8) shell, thus integrating photothermal capability with controlled Zn^2+^ delivery. Under the endogenous acidic tumor microenvironment conditions and exogenous NIR-II laser irradiation, the ZIF-8 shell degrades controllably, resulting in on-demand Zn^2+^ release. This release disrupts intracellular Zn^2+^ homeostasis, together with the LSPR effect of GNS, triggers a massive ROS burst via stimulated mitochondrial ROS production. The resulting oxidative stress induces nuclear and mitochondrial damage, facilitating cytosolic dsDNA accumulation. Zn^2+^ then acts synergistically with cytosolic dsDNA to activate the cGAS-STING pathway and stimulate IFNβ secretion. Simultaneously, photothermal effects combined with oxidative stress induce ICD, releasing DAMPs such as CRT, HMGB1, and ATP. The combination of ICD induction and cGAS-STING activation promotes DCs maturation, antigen presentation, and enhanced CD4^+^/CD8^+^ T cell infiltration, ultimately contributing to immune memory establishment. The activated CD8^+^ T cells further mediate tumor killing, thereby orchestrating the innate-to-adaptive immune response and establishing a cancer-immunity cycle. Together, this approach achieves synergistic NIR-II photothermal therapy and immunotherapy, providing a robust cancer therapy strategyFig. 1.

**Fig. 1:**
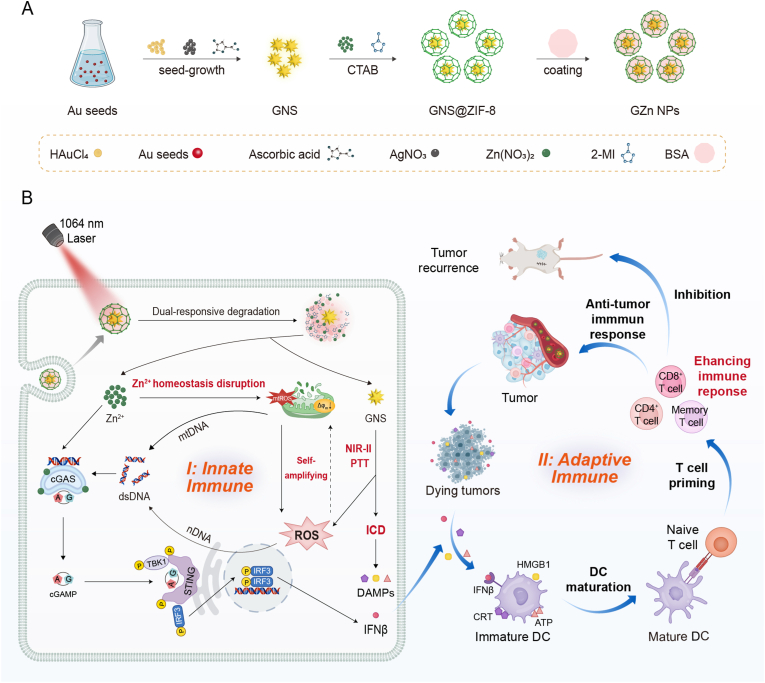
(A) Preparation of the Au-Zn bimetallic STING nanomodulators (GZn NPs) and (B) the therapeutic mechanism of the nanomodulators orchestrating the innate-to-adaptive immune response and establishing a cancer-immunity cycle for amplifying NIR-II photothermal-immunotherapy.

## 2 Results and discussion

### 2.1 Fabrication and characterization of GZn NPs

**Fig. 2:**
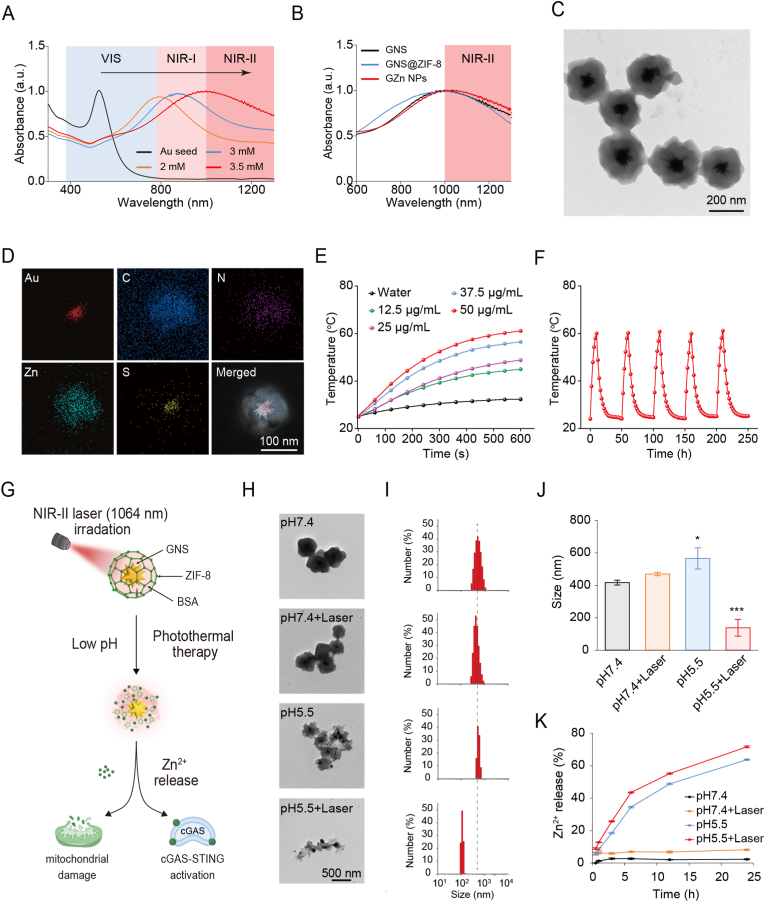
**Characterization of GZn NPs.** (A) UV-Vis-NIR absorbance spectra of Au seed and GNS with different concentrations of AgNO_3_. (B) UV-Vis-NIR absorbance spectra of GNS, GNS@ZIF-8, and GZn NPs. (C) Representative TEM images of GZn NPs. Scale bar: 200 nm. (D) Element mapping of GZn NPs. Scale bar: 100 nm. (E) Temperature curves of GZn NPs with different concentrations after 1064 nm laser irradiation. (F) Photothermal stability of GZn NPs after five cycles of laser on/off (10 min for each irradiation). (G) Schematic diagram of responsive degradation of GZn NPs nanomodulators. (H) TEM images of GZn NPs under different conditions. Scale bar: 500 nm. (I) Hydrodynamic diameter of GZn NPs under different conditions. (J) Changes in hydrodynamic diameters of GZn NPs under different conditions (n = 3). (K) Cumulative releasing curves of Zn^2+^ from GZn NPs under different conditions. Data are expressed as the mean ± SD. Statistical significance was assessed by one-way ANOVA (∗p < 0.05, ∗∗∗p < 0.001, versus the pH7.4 group).

To fabricate Au-Zn bimetallic nanomodulators, GNS were first synthesized via a seed-mediated growth method. The absorption peak of gold seeds initially appeared in the visible (VIS) region (Fig. 2A). Markedly, modulation of AgNO_3_ concentration induced a progressive redshift of absorption peak into the NIR-II window, ultimately producing GNS with intense NIR-II absorption crucial for superior efficiency compared to VIS and first near-infrared (NIR-I) windows (Fig. 2A and S1). Subsequently, Zn^2+^ and 2-methylimidazole (2-MI) were coordinated to form ZIF-8, yielding a core-shell structured GNS@ZIF-8, and further modified with bovine serum albumin (BSA) to generate GNS@ZIF-8@BSA (denoted as GZn NPs). Negligible spectral variations between GNS and GZn NPs confirmed minimal optical interference from ZIF-8 and BSA modifications (Fig. 2B). Transmission electron microscopy (TEM) image characterized multi-branched GNS (126.8 nm) transitioning to uniform core-shell GNS@ZIF-8 (294.8 nm) and finally to GZn NP (327.7 nm) (Fig. 2C, S2 and S3A). Zeta potentials shifted from +32.6 mV (GNS@ZIF-8) to −32 mV (GZn NPs) (Fig. S3B). The Fourier transform infrared (FTIR) spectra displayed the characteristic absorption peaks of GNS@ZIF-8 and GZn NPs at 3135, 2926, 1579, 1421, 1307, 1175, 1142, 995, 756, and 680 cm^−1^ (Fig. S4), with new peaks appearing at ∼1650 cm^−1^ and 3300 cm^−1^ for GZn NPs, further confirming the successful coating of BSA, further confirming successful BSA coating, consistent with literature reports [59]. The highly negative surface charge of GZn NPs confers colloidal stability through electrostatic repulsion, as evidenced by maintained size distribution and morphology in physiological media over 7 days (Figs. S5 and S6). To further investigate the crystalline structure of GZn NPs, a high-resolution transmission electron microscopy (HRTEM) image resolved lattices confirming GNS single crystallinity, and the X-ray diffraction (XRD) analysis revealed diffraction peaks corresponding to both ZIF-8 and Au crystal planes (Figs. S7 and S8). X-ray photoelectron spectroscopy (XPS) results confirmed the presence of Zn, C, N, and O as the surface constituent elements of GZn NPs (Fig. S9). Elemental mapping further verified that GZn NPs consist of Au, Zn, S, N, and C (Fig. 2D), consistent with their designed composition. The Au: Zn molar ratio in GZn NPs is approximately 1:3.45. These results collectively demonstrate the successful synthesis of colloidally stable GZn NPs with prominent NIR-II absorption, establishing their significant potential for PTT in the NIR-II window.

Building on the established potential of GNS for NIR-II PTT, we further elucidated the GNS photothermal performance. As demonstrated in Figure S10-S13, GNS exhibit excellent photothermal properties under NIR-II laser irradiation. Then, we systematically evaluated the GZn NPs' photothermal performance. Results revealed significant concentration-and power-dependent heating profiles, with 50 ppm GZn NPs achieving a temperature elevation to 61.1 °C (Fig. 2E and S14). Further to this, under the five laser on/off cycles, GZn NPs display exceptional photothermal stability (Fig. 2F). The photothermal conversion efficiency of GZn NPs was calculated to be 48.5% (Fig. S15). Furthermore, the photothermal effect of GZn NPs was evaluated in vivo. After intravenous injection, 4T1 tumor-bearing mice were irradiated with an NIR-II laser, and the temperature at the tumor site was monitored in real time by infrared thermal imaging (Fig. S16). These results demonstrate that GZn NPs, derived from GNS cores, retain robust photothermal performance in the NIR-II window. Given the established acid-lability of ZIF-8, the photothermal effect from GNS may further accelerate the degradation of the Au-Zn bimetallic nanomodulators, facilitating Zn^2+^ release. To further investigate, we devised GZn NPs degradation under dual stimuli: endogenous acidic microenvironment (pH 5.5) and exogenous NIR-II laser irradiation (Fig. 2G). TEM imaging revealed negligible morphological changes under physiological conditions (pH 7.4) or laser-only exposure, whereas dual stimulation triggered significant fragmentation of GZn NPs (Fig. 2H). Additionally, hydrodynamic size measurements showed particle aggregation at pH 5.5 (565.7 nm), but dramatic size reduction to 138.2 nm under combined low pH and irradiation conditions (pH 5.5+Laser) (Fig. 2I and J), indicating stimulus-responsive disintegration. Given that acidic microenvironment and NIR-II irradiation are pivotal triggers for GZn NPs degradation, we further evaluated the controlled release of Zn^2+^. Acidic conditions (pH 5.5) induced 63.7% cumulative Zn^2+^ release, which escalated to 71.7% with combined laser irradiation (pH 5.5+Laser). In contrast, minimal Zn^2+^ release was observed under neutral conditions (pH 7.4) or with laser exposure alone (Fig. 2K).

Taken together, the engineered Au-Zn bimetallic nanomodulators maintain structural integrity under physiological conditions but undergo disintegration under dual stimulation of acidic microenvironment and NIR-II laser irradiation. The substantial Zn^2+^ release from degraded GZn NPs can thus potently enhance antitumor immunity through multiple pathways. This synergistic integration of NIR-II PTT and immunotherapy establishes GZn NPs as an effective platform for combinatorial tumor treatment.

### 2.2 NIR-II laser irradiation disrupts Zn^2+^ homeostasis, triggering a self-amplifying ROS burst

**Fig. 3:**
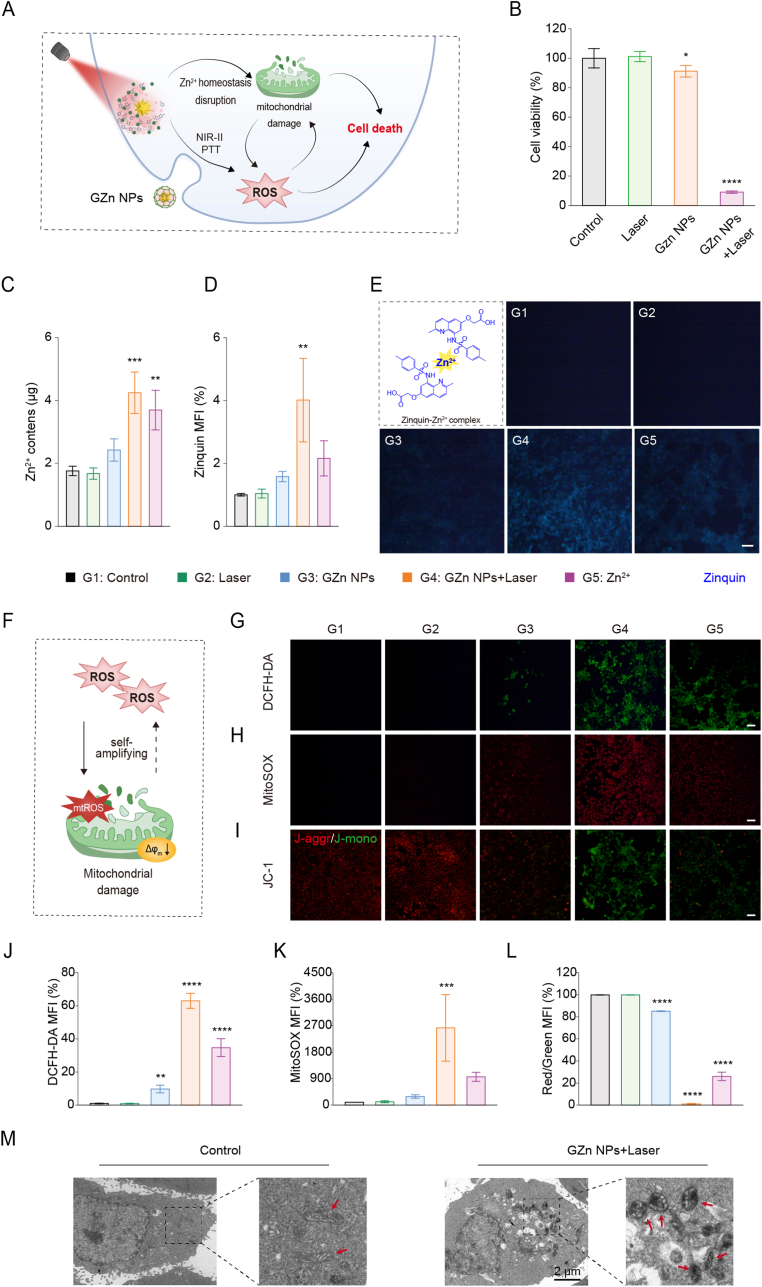
**NIR-II laser irradiation and disruption of Zn^2+^ homeostasis interfere ROS burst**. (A) Schematic illustration for degradable GZn NPs-induced cell death. (B) Cell viability treated with different samples for 24 h (n = 5). (C) Intracellular Zn^2+^ concentration by inductively coupled plasma mass spectrometry (ICP-MS) (n = 3). (D) Quantification analysis and (E) representative images by the Zinquin staining. Scale bar: 50 μm (n = 3). (F) Schematic illustration for ROS burst and mitochondrial damage mediated by GZn NPs with NIR-II laser irradiation treatment. Representative fluorescence images and quantification by the DCFH-DA staining (G, J), Mito Sox Red staining (H, K), and JC-1 staining (I, L) (n = 3). Scale bars 50 μm. (M) Mitochondrial morphology with or without GZn NPs and laser treatment. Scale bar: 2 μm. Data are expressed as the mean ± SD. Statistical significance was assessed by one-way ANOVA (∗∗p < 0.01, ∗∗∗p < 0.001, ∗∗∗∗p < 0.0001, versus the Control group).

Zn^2+^ homeostasis imbalance, which is caused by overloading of Zn^2+^, has, of late, been recognized as playing a vital role in the development, invasion, and metastasis of various tumors [48,49,60]. Capitalizing on potent photothermal efficacy and stimuli-triggered degradation with concomitant Zn^2+^ release, GZn NPs enable amplifying of ROS burst, which leads to effective tumor ablation. (Fig. 3A). Cell viability assays revealed negligible cytotoxicity from GZn NPs alone (Fig. S17), whereas the combined effect of NIR-II photothermal effect and laser-triggered Zn^2+^ liberation induces pronounced tumor cell death (Fig. 3B and S18). Cellular uptake of coumarin 6-labeled GZn NPs nanoagonists in 4T1 cells was assessed by confocal laser scanning microscope (CLSM) and flow cytometry. Both methods demonstrated a rapid intracellular endocytosis behavior with fluorescence intensity increasing over incubation time (Fig. S19). Quantitative inductively coupled plasma mass spectrometry (ICP-MS) analysis demonstrated a substantial elevation of intracellular Zn^2+^ level in GZn NPs + Laser group in contrast with the free Zn^2+^ group and GZn NPs alone (Fig. 3C). Within Zinquin fluorescence probing, GZn NPs + Laser group showed the most intense blue fluorescence (representing free Zn^2+^) compared to other treatment groups, which aligns with the above findings, indicating that GZn NPs potentiate Zn^2+^ accumulation in tumor cells and disrupts Zn^2+^ homeostasis (Fig. 3D and E).

Under NIR-II laser irradiation, GZn NPs generate LSPR and excite hot electrons, contributing to ROS production [[31], [32], [33], [34]]. Concurrently, intracellular Zn^2+^ overload impairs mitochondrial ETC components, stimulating mitochondrial ROS (mtROS) production and upregulation [45,46,[52], [53], [54], [55], [56]]. In essence, ROS generated via the mitochondrial respiratory chain can further damage mitochondria, establishing a self-amplifying ROS cycle that results in severe mitochondrial damage mediated by the Au-Zn bimetallic nanomodulators (Fig. 3F) [[61], [62], [63], [64], [65]]. We next assessed the ROS levels in the intracellular and mitochondrial by DCFH-DA and MitoSOX probe, and aimed to confirm the ROS burst. We observed upregulation in the expression of ROS in the GZn NPs + Laser group versus minimal signal with GZn NPs alone (Fig. 3G and J). Additionally, an obvious enhancement of mtROS level was displayed in GZn NPs + Laser treated groups, indicating the unquestionable production of mtROS (Fig. 3H and K). The limited ROS induction observed with GZn NPs alone is likely due to partial degradation of ZIF-8 and the consequent minor release of Zn^2+^. In contrast, when combined with NIR-II laser irradiation, GZn NPs promote a self-amplifying ROS cycle, leading to a pronounced ROS burst. Importantly, this ROS amplification cycle is Zn^2+^-dependent, as evidenced by a marked reduction in both the Zn^2+^ probe fluorescence signal and intracellular ROS levels following treatment with the specific Zn^2+^ chelator TPEN (Fig. S20). Membrane potential assessment (JC-1 probe) showed dominant green fluorescence (depolarized membranes) and diminished red signal in GZn + Laser group among all the treatment groups (Fig. 3I and L). Besides, Bio-TEM analysis revealed that mitochondria in the control group maintained a largely normal morphology. Conversely, tumor cells treated with GZn NPs plus laser irradiation exhibited a high prevalence of swollen mitochondria (Fig. 3M). These findings further confirm the ROS burst, which is engaged in mitochondrial damage.

Together, under NIR-II irradiation, the Au-Zn bimetallic nanomodulators effectively disrupt intracellular Zn^2+^ homeostasis, thereby causing a ROS burst. This process provokes severe mitochondrial damage, facilitating the release of dsDNA into the cytosol. The cytosolic dsDNA, in synergy with Zn^2+^, then activates the cGAS-STING pathway, realizing the combined potential of NIR-II photothermal therapy and immunotherapy.

### 2.3 Coordinated regulation of dsDNA and Zn^2+^ levels to activate the cGAS-STING pathway

**Fig. 4:**
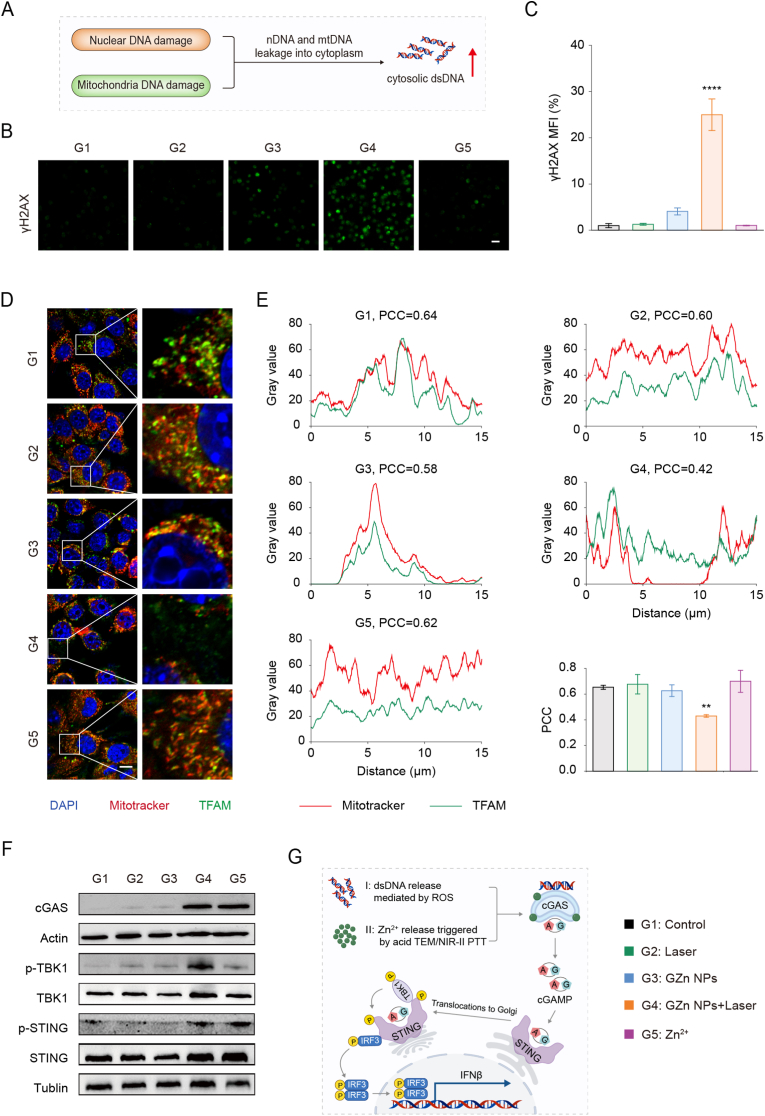
**GZn NPs nanomodulators induce cGAS-STING pathway activation.** (A) Schematic illustration of GZn NPs with NIR-II laser irradiation treatment-triggered simultaneous mitochondrial and nuclear damage and intracellular dsDNA accumulation. (B) Fluorescence images and (C) quantification of the DNA damage marker of γH2AX. Scale bar: 20 μm (n = 3). (D) CLSM images stained with TFAM (mtDNA, green), mitotracker (mitochondrial, red), and DAPI (nucleus, blue). Scale bar: 10 μm. (E) Co-localization analysis and the calculated Pearson correlation coefficient (PCC) from Fig. 4D. (F) cGAS-STING protein expression treated with different samples by western blot. (G) Schematic illustration for the activation of the cGAS-STING pathway through GZn NPs with NIR-II laser irradiation treatment. Data are expressed as the mean ± SD. Statistical significance was assessed by one-way ANOVA (∗∗p < 0.01, ∗∗∗∗p < 0.0001, versus the Control group).

Our previous findings demonstrated that NIR-II laser irradiation triggers GZn NPs degradation, inducing Zn^2+^ release that disrupts Zn^2+^ homeostasis and leads to ROS bursts, thereby causing severe mitochondrial damage. On the other hand, elevated ROS levels were able to induce nDNA double-strand breaks [66]. We postulate that the damage to nuclear and mitochondrial may facilitate cytosolic release of nDNA and mtDNA, as modeled conceptually in Fig. 4A. To investigate nuclear damage, immunofluorescence staining revealed a marked upregulation of γH2AX expression in the GZn NPs + Laser group compared to other treatments (Fig. 4B and C, and S21). Given that γH2AX is a sensitive biomarker for DNA double-strand breaks, these findings confirm substantial nuclear genotoxic stress, which is a necessary precondition for the potential release of nDNA into the cytosol. To further investigate, immunofluorescence co-localization assays employing mitochondrial transcription factor A (TFAM) and MitoTracker co-staining were performed to monitor mtDNA release. In the GZn NPs + Laser group, the green fluorescence of TFAM showed poor colocalization with the red mitochondrial fluorescence (Fig. 4D and S22). The reduced Pearson's correlation coefficient (PCC) indicates mitochondrial disruption, a state conducive to mtDNA release (Fig. 4E). Together, these results demonstrate that the treatment inflicts severe damage on both nuclear and mitochondrial DNA, creating conditions favorable for the accumulation of cytosolic dsDNA. These results together with the aforementioned findings (Fig. 3G, J, and 3K), bring a potential for upregulation of the cytosolic dsDNA.

Cytosolic dsDNA, including nDNA and mtDNA, is a widely acknowledged endogenous signal that activates the cGAS-STING pathway. Zn^2+^ has recently been identified as crucial for amplifying cGAS phase separation and enzymatic activity [50,51]. To determine whether the coordinated regulation of dsDNA and Zn^2+^ levels owing to GZn NPs + Laser treatment could activate the cGAS-STING signaling pathway, we analyzed upstream cGAS expression and downstream phosphorylation events (p-STING and p-TBK1) via western blot. The results showed significantly upregulated cGAS expression and enhanced phosphorylation of STING and TBK1 in the GZn NPs + Laser group compared to all other groups, confirming the activation of the cGAS-STING signaling pathway (Fig. 4F and G). Quantitative PCR (qPCR) analysis revealed that treatment with GZn NPs + Laser significantly upregulated the mRNA expression of IFNB1 and ISG56, two key genes associated with type I interferon signaling. Furthermore, the expression of pro-inflammatory cytokines, including TNFα, IL-6, and CXCL10, was also elevated following GZn NPs treatment (Fig. S23).

In summary, GZn NPs combined by NIR-II laser irradiation appear to be an indispensable STING nanomodulator and amplifier of cGAS-STING pathway activation. As a fundamental innate immune signaling axis, this pathway bridges innate sensing to adaptive immunity by enhancing DCs cross-presentation and CD8^+^ T cell priming, thereby establishing the foundation for systemic anti-tumor immunity.

### 2.4 NIR-II PTT and oxidative stress-driven ICD induction

**Fig. 5:**
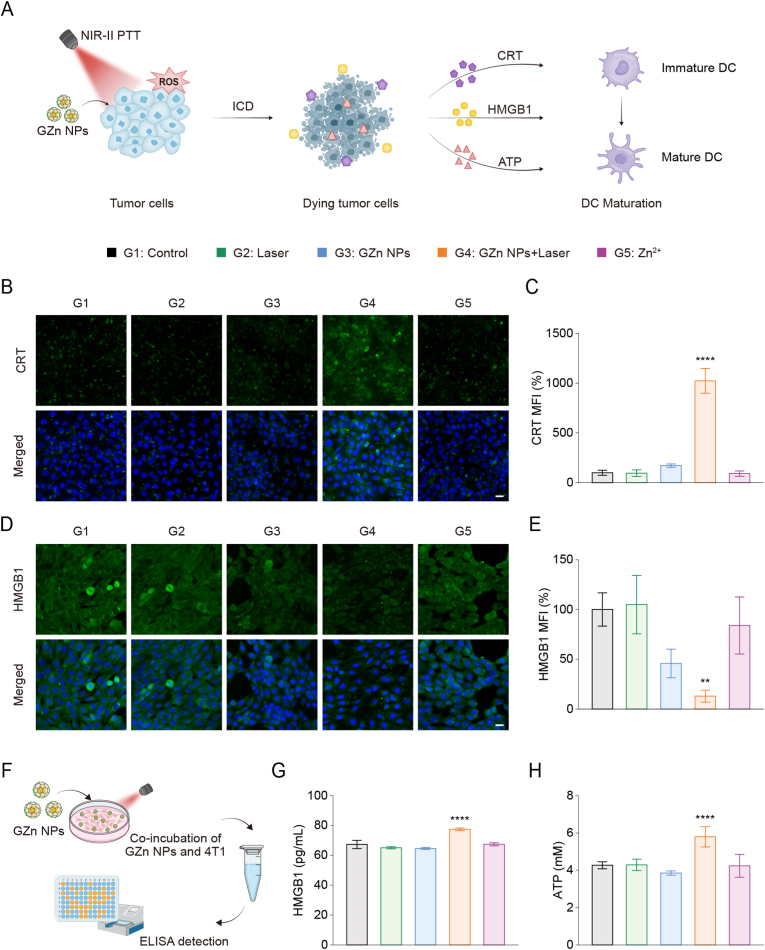
**Evaluation of ICD induction.** (A) Schematic illustration for GZn NPs with NIR-II laser irradiation treatment-induced ICD that is characterized by CRT exposure, HMGB1 release, and ATP secretion. (B) Fluorescence images and (C) quantification of calreticulin (CRT) expression (n = 3). Scale bar: 20 μm. (D) Fluorescence images and (E) quantification of high mobility group box protein B1 (HMGB1) expression (n = 3). Scale bar: 20 μm. (F) The diagram of the ELISA experiment. (G) Extracellular HMGB1 levels after indicated treatment (n = 3). (H) Analysis of ATP levels after indicated treatment (n = 3). Data are expressed as the mean ± SD. Statistical significance was assessed by one-way ANOVA (∗∗p < 0.01, ∗∗∗∗p < 0.0001, versus the Control group).

Immunogenic cell death (ICD), a specific cell death modality triggered by the NIR-II photothermal effects and ROS burst, is characterized by the release of DAMPs. These DAMPs, including CRT, HMGB1, and ATP, act as 'eat-me' signals that promote DC maturation and amplify immune responses (Fig. 5A). To delve into whether GZn NPs combined with NIR-II laser irradiation could initiate ICD, we investigated DAMPs release in vitro. Utilizing immunofluorescence analysis, we observed that CRT translocated to the plasma membrane following GZn NPs + Laser treatment, accompanied by a significant increase in fluorescence intensity, which is consistent with both our expectations and previously reported findings (Fig. 5B). Quantitative analysis demonstrated a 10.23-fold increase in intracellular CRT levels compared to controls based on mean fluorescence intensity (MFI) measurements (Fig. 5C). For the late-stage ICD biomarker HMGB1, which functions as an extracellular cytokine, attenuated intracellular green fluorescence in the GZn NPs + Laser group demonstrated active efflux, with intracellular HMGB1 levels decreasing by 87.1% compared to control (Fig. 5D and E). In addition, extracellular ATP additionally functions as a 'find-me' signal that recruits and activates DCs. The immunogenic substances, including HMGB1 and ATP, were measured by ELISA assays, respectively (Fig. 5F). ELISA quantification further confirmed elevated extracellular release of both HMGB1 and ATP post-treatment (Fig. 5G and H).

Collectively, these results demonstrate that the NIR-II photothermal effect of GZn NPs, combined with NIR-II laser irradiation, amplifies the ROS burst and ultimately induces ICD. It is noteworthy to mention that the cGAS-STING pathway plays a critical role in antitumor immunity. Building on our previous findings that GZn NPs could effectively activate the cGAS-STING pathway to produce type I IFNs and elicit ICD-associated DAMPs release, this dual immunostimulatory mechanism is poised to orchestrate the transition from innate to adaptive immunity, thereby amplifying the efficacy of tumor immunotherapy.

### 2.5 cGAS-STING activation and ICD induction drive DC maturation, potentiating robust antitumor immunity

**Fig. 6:**
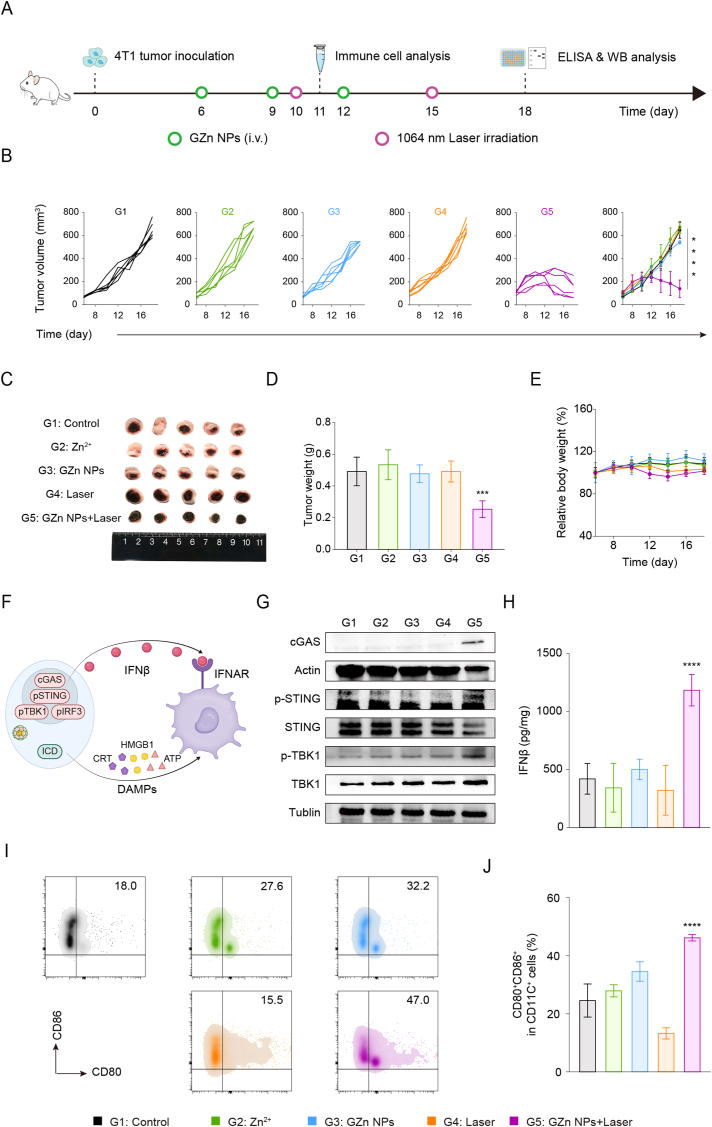
**In vivo anti-tumor efficacy evaluation of nanomodulators.** (A) Schematic illustration of animal experiments. (B) Tumor growth curves following various treatments (n = 5). (C) Pictures of tumor dissected, (D) tumor weight, and (E) mice relative body weight after various treatments (n = 5). (F) Schematic illustration of DCs maturation by cGAS-STING activation and ICD induction. (G) Western blot analysis of tumor tissues derived from Fig. 6C with different treatments. (H) Secretion of IFNβ in randomly selected tumor samples derived from Fig. 6C was detected using an ELISA kit (n = 3). (I) Flow cytometry plots and (J) corresponding quantification analyses of mature DCs in tumor-draining lymph nodes (TDLNs). Data are expressed as the mean ± SD. Statistical significance was assessed by one-way ANOVA (∗∗∗p < 0.001, ∗∗∗∗p < 0.0001, versus the Control group).

Activation of the cGAS-STING pathway enhances DCs cross-presentation through IFNβ secretion, while ICD promotes DC maturation via the release of DAMPs. Motivated by this dual immunostimulatory mechanism, we hypothesized that coordinated activation of the cGAS-STING pathway and induction of ICD could synergistically drive DC maturation, promote tumor-specific CD8^+^ T cells activation, and bridge innate and adaptive immune responses. Based on this premise, we next investigated whether the nanomodulators could robustly activate the immune system and achieve potent tumor suppression in vivo. Following a predetermined experimental schedule, we evaluated the in vivo tumor suppression and immune activation efficacy of the nanomodulators (Fig. 6A). Tumor growth curves demonstrated the most pronounced suppression of tumor growth in mice treated with GZn NPs plus laser (Fig. 6B). This enhanced antitumor effect was further supported by digital images of the resected tumors and subsequent quantitative analysis (Fig. 6C and D). Spleen weight measurement revealed reduced splenomegaly in the GZn NPs + Laser group (Fig. S24). Hematoxylin and eosin (H&E) staining of tumor sections demonstrated that tumor cells in GZn NPs + Laser group clearly caused reduced nuclear density with obvious damage and necrosis in stark contrast to the numerous intact nuclei observed in controls, further evidencing potent tumor inhibition (Fig. S25). Throughout the treatment period, body weight remained stable, indicating negligible systemic toxicity (Fig. 6E).

We also examined the in vivo biosafety of GZn NPs through histopathological and hematological analyses. Administration of the nanomodulator did not induce notable body weight fluctuations or cause pathological damage in major organs, as confirmed by H&E staining (Figs. S26 and S27). Furthermore, no apparent abnormalities were detected in the blood routine examination and blood biochemical analyses (Figs. S28 and S29). Additionally, in vitro hemolysis assays confirmed the blood biocompatibility of GZn NPs. As seen in Fig. S30, almost negligible hemoglobin release occurred from red blood cells treated with GZn NPs at concentrations of 100 μg/mL. All the data demonstrated the excellent biocompatibility of nanomodulators both in vitro and in vivo.

Considering our observations that NIR-II laser irradiation and the acidic tumor microenvironment could trigger nanomodulator degradation, subsequently Zn^2+^ release leading to the intracellular disruption of Zn^2+^ homeostasis and ROS burst. Then, we sought to determine whether IFNβ secretion derived from the activation of the cGAS-STING pathway in vivo could promote DCs maturation and further elicit potent antitumor immunity (Fig. 6F). Western blot analysis confirmed that GZn NPs + Laser treatment robustly activated the cGAS-STING pathway, as evidenced by significantly elevated levels of cGAS, p-STING, and p-TBK1 (Fig. 6G). Moreover, ELISA confirmed elevated intratumoral IFN-β secretion in this group (Fig. 6H). Activation of the cGAS-STING pathway enhances the maturation of DCs via IFNβ secretion, thereby activating the proliferation of naive T cells and evoking an adaptive immune response. Subsequent flow cytometry analysis of tumor-draining lymph nodes (TDLNs) revealed a 1.9-fold higher proportion of matured DCs (CD80^+^CD86^+^) in the GZn NPs + Laser group compared to the control (Fig. 6I and J), validating that the STING nanomodulators effectively combine NIR-II photothermal therapy with immunotherapy.

Collectively, the aforementioned findings illustrated that the STING nanomodulators combined with NIR-II laser irradiation provoke effective antitumor immunity in vivo by activating the cGAS-STING pathway within tumor regions and promoting DCs maturation in TDLNs, effectively suppressing tumor growth. Extensive evidence indicates that mature DCs, as pivotal immunomodulatory hubs, exhibit the exceptional capacity to activate effector T cells and memory T cells while orchestrating innate immunity and adaptive immunity.

### 2.6 Enhanced DC maturation orchestrates the innate-to-adaptive immune response and establishes a cancer-immunity cycle

**Fig. 7:**
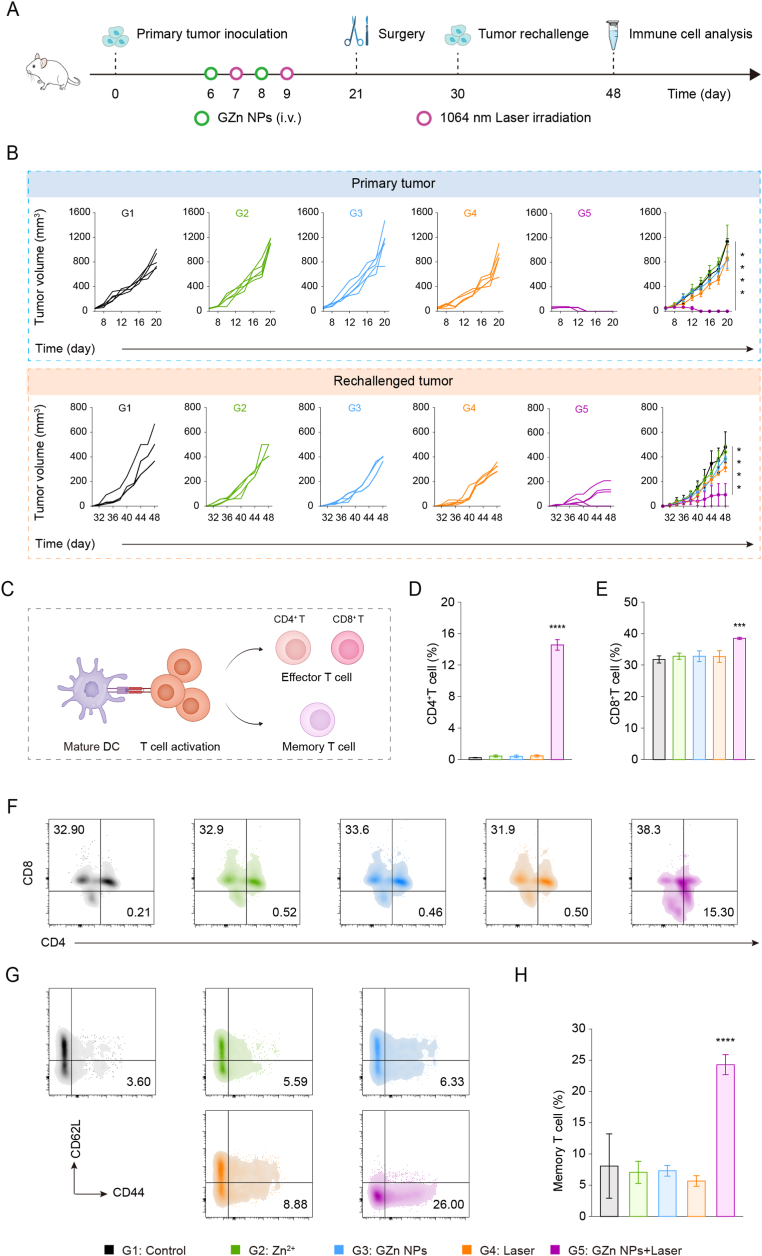
**Prevention of tumor recurrence through immunological memory.** (A) Schematic illustration of animal experiments. (B) The primary tumor and the rechallenged tumor growth curves following various treatments (n = 5). (C) Schematic illustration of immune responses by GZn NPs with NIR-II laser irradiation treatment. (D, E) Quantification analyses and (F) flow cytometry plots of CD4^+^ and CD8^+^ T cells in the rechallenged tumor (n = 3). (H) Flow cytometry plots and (I) corresponding quantification analyses of memory T cells in spleen (n = 3). Results are presented as means ± SD. Statistical significance was calculated using a one-way ANOVA (∗∗∗∗p < 0.0001, versus the Control group).

The effective activation of immune responses by GZn NPs combined with NIR-II laser irradiation prompted further evaluation of their ability to elicit adaptive immunity against tumor recurrence and establish long-term immunological memory. For this purpose, a tumor cell rechallenged model was established, and the treatment process was depicted in Fig. 7A. GZn NPs + Laser treatment not only eradicated the primary tumor but also effectively suppressed the growth of rechallenged tumors until the experimental endpoint (day 48), whereas both primary and rechallenged tumors in the control group progressed rapidly (Fig. 7B). These results underscore the potential of this strategy to promote DCs maturation, enhance tumor infiltration of effector T cells, and establish durable memory T cell responses, thereby orchestrating innate to adaptive immunity. Furthermore, to better clarify the prevention mechanism of rechallenged tumors, we examined tumor-infiltrating helper T cells (CD4^+^ T cells), cytotoxic T lymphocytes (CD8^+^ T cells), and memory T cells (CD44^high^ CD62L^low)^ through FCM analysis (Fig. 7C). The GZn NPs + Laser group exhibited a substantial enhancement of CD4^+^ T cells (14.6%) and CD8^+^ T cells (38.4%) compared to controls (Fig. 7D-F), indicating that the DCs' maturation could increase intra-tumoral infiltration of effector T cells.

Besides, memory T cell populations in splenic tissues were quantified across treatment groups (Fig. 7G). Mice treated with GZn NPs + Laser showed a 3-fold increase in memory T cells, confirming the successful generation of long-term immune memory. Based on these findings, we propose a cancer-immunity cycle wherein infiltrating CD8^+^ T cells directly kill tumor cells. The dying tumor cells may then be captured by DCs, potentially leading to renewed CD8^+^ T cell activation.

Taken together, cGAS-STING pathway activation mediated by GZn NPs plus NIR-II laser irradiation not only exerts potent suppression of primary tumors but also enhances DC maturation, enabling long-term immunosurveillance and inhibition of rechallenged tumors. By establishing the cancer-immunity cycle, this process promotes further effector T-cell infiltration into tumors, ultimately establishing a robust and sustained antitumor immune effect.

## 3 Conclusions

In summary, this study successfully developed a dual-responsive and intelligently designed Au-Zn bimetallic STING nanomodulators (designated as GZn NPs) with stimuli-degradable properties. Under NIR-II laser irradiation and the acidic tumor microenvironment, GZn NPs undergo degradation, accompanied by controlled Zn^2+^ release, which disrupts intracellular Zn^2+^ homeostasis and triggers a ROS self-amplifying ROS cycle. Within this cycle, a ROS burst is facilitated, causing nuclear and mitochondrial damage and promoting the release of nDNA and mtDNA, thereby elevating cytosolic dsDNA levels. The coordinated interplay of Zn^2+^ and dsDNA effectively activates the cGAS-STING pathway, driving abundant IFNβ secretion. Simultaneously, the NIR-II photothermal effects and ROS burst induce ICD, accompanied by the release of DAMPs, including CRT, HMGB1, and ATP. Abundant secretion of IFNβ together with DAMPs release potently stimulates DC maturation, which enhances the infiltration of effector T cells (eg, CD4^+^ and CD8^+^) and supports memory T cell generation. The infiltrated effector T cells further kill tumor cells, which can be captured by DCs, stimulating DC maturation and re-priming T cells, eventually establishing a cancer-immunity cycle. This cyclical process efficiently bridges and orchestrates innate-to-adaptive immune responses, then establishes durable memory T cell responses that validly suppress both primary and recurrent tumors. With excellent biocompatibility, responsive biodegradability, controlled release behavior, and strong immunotherapeutic potential, Au-Zn bimetallic STING nanomodulators, combined with NIR-II laser irradiation, offer a promising strategy that integrates NIR-II photothermal therapy with immunotherapy, representing a robust amplification and combination approach for tumor treatment.

This work demonstrates significant innovation in material design and therapeutic strategy. Beyond the specific Au-Zn system, it serves as a proof-of-concept for a "degradable metalloimmunotherapy platform." The core design principle-using a responsive, biodegradable inorganic matrix to co-deliver bioactive metal ions and mediate physical stimuli-provides a versatile conceptual framework. This strategy can be adapted to other immunomodulatory metals or combined with different therapeutic modalities to target a broader spectrum of immune signaling pathways for tailored and potent anti-tumor therapy. We acknowledge the limitations of this study, including the need for validation in more complex tumor models. Future efforts will focus on enhancing the photothermal conversion efficiency of GZn NPs to enable effective tumor ablation at lower, more clinically relevant power densities. Additionally, the in vivo metabolic pathways and long-term safety profile of GZn NPs are important issues that warrant thorough investigation in our subsequent studies. Despite these challenges, the GZn NPs platform, with its favorable biocompatibility, controlled degradation, and potent immune-activating capability, represents a promising and broadly applicable strategy for synergistic photothermal-immunotherapy.

## CRediT authorship contribution statement

**Qiaolin Wei:** Conceptualization, Formal analysis, Funding acquisition, Investigation, Project administration, Supervision, Validation, Writing – review & editing. **Zirui Zhu:** Data curation, Investigation, Software, Validation, Visualization, Writing – original draft, Writing – review & editing. **Yue Li:** Data curation, Investigation, Software, Validation, Visualization, Writing – original draft, Writing – review & editing. **Siying Sun:** Data curation, Investigation, Validation, Visualization. **Ge Gao:** Investigation, Validation. **Yinghong Wan:** Investigation, Validation. **Yi Hao:** Investigation, Validation. **Jiaying Lei:** Investigation, Validation. **Jiahao Xu:** Investigation, Validation. **Quan Hu:** Resources, Supervision. **Wei Zheng:** Resources, Supervision. **Yong Guo:** Conceptualization, Investigation, Project administration, Supervision, Validation, Writing – review & editing. **Jia-Wei Shen:** Conceptualization, Funding acquisition, Investigation, Project administration, Resources, Supervision, Validation, Writing – review & editing.

## Declaration of competing interest

The authors declare that they have no known competing financial interests or personal relationships that could have appeared to influence the work reported in this paper.

## Data Availability

Data will be made available on request.
