# Supplementary material for: Degradable STING nanomodulators orchestrate the innate-to-adaptive immune response for NIR-II photothermal-immunotherapy via a cancer-immunity cycle
Source: Mater Today Bio. 2026 Mar 14;38:103028. doi: 10.1016/j.mtbio.2026.103028 (PMC13022635; doi:10.1016/j.mtbio.2026.103028)
Supplement: Multimedia component 1 [file mmc1.docx]

Supporting Information

**Degradable STING** **Nanomodulators Orchestrate The Innate-To-Adaptive Immune Response For NIR-II Photothermal-Immunotherapy Via a Cancer-Immunity Cycle**

Qiaolin Wei ^a, b, 1*^ Zirui Zhu ^a, c, 1^, Yue Li ^a, 1^, Siying Sun ^a^, Ge Gao ^a^, Yinghong Wan ^a^, Yi Hao ^a^, Jiaying Lei ^a^, Jiahao Xu ^a^, Quan Hu ^a^, Wei Zheng ^a^, Yong Guo ^a, b*^, Jia-Wei Shen ^a, b*^

^a^Zhejiang Provincial Key Laboratory of Anti-Cancer Chinese Medicines and Natural Medicines, School of Pharmacy, Hangzhou Normal University, Zhejiang 311121, China.

^b^Engineering Laboratory of Development and Application of Traditional Chinese Medicines, Collaborative Innovation Center of Traditional Chinese Medicines of Zhejiang Province, Hangzhou Normal University, Hangzhou, Zhejiang 311121, China.

^c^ College of Life and Environmental Sciences, Hangzhou Normal University, Hangzhou, Zhejiang 311121, China.

Corresponding author at: School of Pharmacy, Hangzhou Normal University, Hangzhou, Zhejiang 311121, China

*E-mail addresses*: weiqiaolin2009@163.com (Q. Wei); guoyong@hznu.edu.cn; shen.jiawei@hotmail.com (J.W. Shen)

^1^ These authors contributed equally to this work

## EXPERIMENTAL SECTION

**Materials**

Sodium citrate was purchased from Shanghai Xian Ding Biotechnology (China). Chloroauric acid (HAuCl_4_·3H_2_O) and ascorbic acid (AA) were purchased from Aladdin (China). Zn(NO_3_)_2_·6H_2_O, silver nitrate (AgNO_3_), and cetyltrimethylammonium bromide (CTAB) were purchased from Sinopharm Chemical Reagent (China). 2-methylimidazole (2-MI) and bovine serum albumin (BSA) were purchased from Macklin (China). DCFH-DA, 4,6-diamidino-2-phenylindole (DAPI), JC-1 staining kit, methylthiazolyldiphenyl-tetrazolium bromide (MTT), Radio Immunoprecipitation Assay (RIPA), and BCA protein assay kit were purchased from Beyotime Inst. Biotech. (China). Calcein-AM and propidium iodide (PI) were purchased from Yeasen Biotechnology (China). Zinquin, MitoSOX Red, and HRP-conjugated AffiniPure Goat Anti-Rabbit IgG H&L were purchased from MedChemExpress (China). The beta Actin Mouse Monoclonal Antibody was purchased from HUABIO (China). The Alpha Tubulin Polyclonal antibody, calreticulin (CRT) Polyclonal antibody, high-mobility group box 1 (HMGB1) Polyclonal antibody, Phospho-Histone H2A.X (Ser139) Recombinant monoclonal antibody, and TFAM Polyclonal antibody were purchased from Proteintech (China). The cGAS antibody, STING antibody, Phospho-STING (Ser365) antibody, TBK1/NAK antibody, and Phospho-TBK1/NAK (Ser172) antibody were purchased from Cell Signaling Technology (U.S.). The Mouse IFNβ ELISA Kit was purchased from MultiSciences Biotech Co., Ltd. The anti-CD45-BV605, anti-CD3-APC, anti-CD11c-FITC, anti-CD80-PE, anti-CD86-APC, anti-CD4-FITC, anti-CD8a-PC7, anti-CD62L-PB450, and anti-CD44-PE were purchased from Biolegend (China).

**Synthesis of gold nanostars (GNS)**

Gold nanostars (GNS) were prepared via seed-mediated growth. The seed solution, obtained by sodium citrate reduction of boiling HAuCl_4_, was stored at 4℃ after cooling and filtration. For GNS synthesis, 100 µL of seed solution was added to 7 mL of 0.25 mM HAuCl_4_ (with 10 µL of 1 M HCl) under 800 rpm stirring, followed by the simultaneous addition of 100 µL AgNO_3_ and 50 µL of 100 mM AA. A rapid color change to blue-black signaled formation. The product was isolated by centrifugation (8000 rpm, 10 min) and re-dispersed in 5×10^-^⁴ M CTAB.

**Synthesis of core-shell GNS@ZIF-8@BSA (GZn NPs)**

The synthesis of GZn NPs involved a sequential solution mixing process. First, 3 mL of CTAB (5×10^-^⁴ M) was combined with 3 mL of 1.32 M 2-MI under stirring for 5 minutes. Subsequently, 3 mL of 24 mM Zn(NO₃)₂·6H₂O and 3 mL of GNS were introduced, followed by another 5 minutes of stirring and a 3 h incubation without disturbance. The particles were isolated via centrifugation (6000 rpm, 5 min), washed with dH_2_O, and redispersed in water. Finally, the preparation was coated with 10 mL of BSA under overnight shaking to improve its biosafety.

**Material characterization**

Fine morphology of the samples was observed by transmission electron microscopy (HITACHI, HT7700, Japan). Morphology and element mapping of GZn NPs were recorded by TEM (FEI, Tecnai G2 F20 S-TWIN, America). UV-Vis spectra were recorded on a spectrometer (SHIMADZU, UV-2600, Japan). The dynamic light scattering size and zeta potential of GZn NPs were measured using a Zetasizer Nano Instrument (Malvern Panalytical, Zetasizer Lab, England). The released amounts of Zn^2+^ ions were quantified by using inductively coupled plasma optical emission spectrometry (FPI, ICP-5000, China). Elemental composition of GZn NPs was analyzed by X-ray photoelectron spectroscopy (Thermo Scientific, Nexsa G2, USA). FT-IR spectra were obtained using the FT-IR spectrometer (HORIBA, HYPERION II, Japan)

**Stability Measurement of GZn NPs**

GZn NPs nanomodulators were dispersed in 1 mL of different solutions (Water, DMEM, and DMEM+10% FBS), and their size was measured by DLS for 7 days. TEM images were also captured after being saved for 7 days.

**Measurement of the photothermal properties**

To assess the photothermal performance of the nanomodulators, GNS and GZn NPs (0, 12.5, 25, 37.5, and 50 μg/mL) at different concentrations were placed in a quartz cell and exposed to a 1064 nm laser at a power density of 2.0 W/cm^2^ for 600 s. Additionally, the GNS and GZn NPs aqueous solution was exposed to irradiation under a 1064 nm laser with different power densities (0.5, 1.0, 1.5, and 2.0 W/cm^2^) for 600 s, respectively. To evaluate the photothermal stability of GNS and GZn NPs, an aqueous solution of GNS and GZn NPs was exposed to a 1064 nm laser at a power density of 2.0 W/cm^2^ for 10 min. This process was then repeated 5 times, with the laser being turned off and the solution allowed to cool to ambient temperature between each repetition. Temperature changes were recorded after each repetition.

To evaluate the photothermal conversion efficiency of GZn NPs, the GZn NPs aqueous solution with the concentration of Au 50 μg/mL was exposed to the irradiation under 2.0 W/cm^2^ 1064 nm laser for 10 min. Subsequently, the solutions cooled down naturally. The photothermal conversion efficiency (η) can be calculated according to the eq (1)

$$\begin{aligned} \eta=\frac{hs\left( T_{max}-T_{suur} \right)-Q_{dis}}{I\left( 1-{10}^{-A_{1064}} \right)}\#\left( 1 \right) \end{aligned}$$

The T_max_ (K) means the equilibrium temperature, T_surr_ (K) is the ambient temperature of the surroundings. The Q_dis_ is the heat loss from light absorbed by the container, and it is calculated to be approximately equal to 0 mW. I (W) represents incident laser power (2.0 W/cm^2^), and A_1064_ is the absorbance of samples at 1064 nm. Where h (W/cm^2^/K) means heat transfer coefficient, s (cm^2^) represents the surface area of the container. The hs is calculated using the following eq (2)

$$\begin{aligned} \tau_{s=\frac{m_{D}c_{D}}{hs}}\#\left( 2 \right) \end{aligned}$$

Where τ_s_ is the sample system time constant, m_D_ and c_D_ are the mass (2 g) and heat capacity (4.2 J/g) of the solvent.

**In vitro dual-responsive degradation and Zn^2+^ release**

The obtained GZn NPs were placed in a dialysis bag and immersed in 30 mL of PBS with different pH values (pH 7.4 and pH 5.5) in tubes with or without laser irradiation. The tube was shaken at 150 rpm (37℃), and supernatant (3 mL) was removed and replaced by the same amount of fresh PBS solution at different time intervals. Samples were irradiated for 10-minute cycles (laser ON/OFF) at specified time points (0, 1, 3, 6, 12, 24 h). Lastly, the concentrations of Zn^2+^ were measured by ICP-MS.

**Cell Culture**

4T1 murine breast tumor cells were cultured in Dulbecco's Modified Eagle Medium (DMEM, Meilunbio) containing 1% antibiotics (100 µg/mL streptomycin and 100 µg/mL penicillin) and 10% fetal calf serum (FBS, Meilunbio), respectively. All cells were maintained in a cell incubator at 37℃ under 5% CO_2_.

**Cell Viability**

4T1 cells were treated with various groups: (1) Control, (2) Laser, (3) GZn NPs, and (4) GZn NPs+Laser, for 2 h, respectively. The concentrations of GZn NPs were 12.5 μg/mL of Au. The concentration of Zn^2+^ was 3 μg/mL. Following 6 h of incubation, the group of Laser and GZn NPs+Laser was irradiated by the NIR-II laser lasting 10 min. Next, cells were incubated in an incubator for 18 h. Then, cell viability was performed via MTT reagent, and 570 nm absorbance was obtained by microplate reader (Thermo Fisher Scientific, Multiskan FC, America).

**Living/Dead Cell Staining Assay**

4T1 cells were treated with various groups: (1) Control, (2) Laser, (3) GZn NPs, and (4) GZn NPs+Laser, for 2 h, respectively. The concentrations of GZn NPs were 12.5 μg/mL of Au. The concentration of Zn^2+^ was 3 μg/mL. Following 6 h of incubation, the group of Laser and GZn NPs+Laser was irradiated by the NIR-II laser lasting 10 min. Next, cells were incubated in an incubator for 18 h. Then, the cells were stained with Calcein-AM (2 μM) and PI (4 μM) and then imaged with Inverted Biological Microscope (Optec, BDS400, China).

**Evaluation of cellular uptake**

4T1 tumor cells were seeded into a 35-mm dish and cultured overnight to ~80% confluence. Then, the cells were incubated with fresh medium containing coumarin 6-labeled GZn NPs for 0, 2, 4, and 6 h, respectively. For imaging, the cells were washed three times with PBS and fixed with 4% paraformaldehyde. After that, the cell nuclei were stained with DAPI and further analyzed by CLSM. For flow cytometric analysis, the cells were washed three times with PBS and detached using a trypsin-EDTA solution, neutralized with complete medium, centrifuged at 300 × g for 5 min, and fixed with 4% paraformaldehyde. The resulting cell pellets were resuspended in PBS for further analysis by flow cytometric analysis.

**Detection of intracellular Zn^2+^ release by inductively coupled plasma-mass spectrometry (ICP-MS) and Zinquin**

ICP-MS: 4T1 cells were treated with various groups: (1) Control, (2) Laser, (3) GZn NPs, (4) GZn NPs+Laser, and (5) Zn^2+^ for 2 h, respectively. The concentrations of GZn NPs were 12.5 μg/mL of Au. The concentration of Zn^2+^ was 3 μg/mL. Following 2 h of incubation, the group of Laser and GZn NPs+Laser was irradiated by the NIR-II laser lasting 10 min. Next, cells were incubated in an incubator for 2 h. Then, the cells were washed three times with phosphate buffer solution (PBS). Subsequently, the cells were trypsinized, counted, and pelleted. The cell pellets were then completely digested with concentrated nitric acid (HNO₃) using a heating block. Finally, the concentrations of Zn^2+^ in collected cells were measured by ICP-MS.

Zinquin: 4T1 cells were treated with various groups: (1) Control, (2) Laser, (3) GZn NPs, (4) GZn NPs+Laser, and (5) Zn^2+^ for 2 h, respectively. The concentrations of GZn NPs were 12.5 μg/mL of Au. The concentration of Zn^2+^ was 3 μg/mL. The group of Laser and GZn NPs+Laser was irradiated by NIR-II laser lasting 10 min. Next, cells were incubated in an incubator for 2 h. Then, the cells were stained with Zinquin and then imaged with Inverted Biological Microscope (Optec, BDS400, China).

**Intracellular ROS detection**

4T1 cells were treated with various groups: (1) Control, (2) Laser, (3) GZn NPs, (4) GZn NPs+Laser, and (5) Zn^2+^ for 2 h, respectively. The concentrations of GZn NPs were 12.5 μg/mL of Au. The concentration of Zn^2+^ was 3 μg/mL. The group of Laser and GZn NPs+Laser was irradiated by NIR-II laser lasting 10 min. Next, cells were incubated in an incubator for 2 h. Then, the cells were stained with DCFH-DA and then imaged with Inverted Biological Microscope (Optec, BDS400, China).

**Detection of Intracellular ·O_2_^-^ Production**

4T1 cells were treated with various groups: (1) Control, (2) Laser, (3) GZn NPs, (4) GZn NPs+Laser, and (5) Zn^2+^ for 2 h, respectively. The concentrations of GZn NPs were 12.5 μg/mL of Au. The concentration of Zn^2+^ was 3 μg/mL. The group of Laser and GZn NPs+Laser was irradiated by NIR-II laser lasting 10 min. Next, cells were incubated in an incubator for 2 h. Then, the cells were stained with MitoSOX Red and then imaged with Inverted Biological Microscope (Optec, BDS400, China).

**Mitochondrial Membrane Potential (MMP) Detection**

4T1 cells were treated with various groups: (1) Control, (2) Laser, (3) GZn NPs, (4) GZn NPs+Laser, and (5) Zn^2+^ for 2 h, respectively. The concentrations of GZn NPs were 12.5 μg/mL of Au. The concentration of Zn^2+^ was 3 μg/mL. The group of Laser and GZn NPs+Laser was irradiated by NIR-II laser lasting 10 min. Then, the cells were stained with JC-1 and then imaged with Inverted Biological Microscope (Optec, BDS400, China).

**Mitochondrial morphological change**

The adherent cells were collected, fixed overnight at 4℃ with 2.5% glutaraldehyde, and then rinsed three times with PBS. Subsequently, they were fixed in 1% osmic acid for 1 hour. Following dehydration through a graded ethanol series, the samples were embedded in acrylic resin. Ultrathin sections were stained with uranyl acetate and lead citrate, and examined using TEM (HITACHI HT7800, Japan).

**DNA damage detection of 4T1 cells in vitro**

4T1 cells were treated with various groups: (1) Control, (2) Laser, (3) GZn NPs, (4) GZn NPs+Laser, and (5) Zn^2+^ for 2 h, respectively. The concentrations of GZn NPs were 12.5 μg/mL of Au. The concentration of Zn^2+^ was 3 μg/mL. The group of Laser and GZn NPs+Laser was irradiated by NIR-II laser lasting 10 min. Next, cells were incubated in an incubator for 2 h. Then, the cells were washed three times with PBS and fixed with 4% paraformaldehyde. Then, cells were treated with 0.3% Triton X-100 for 10 min and sealed with 5% BSA at room temperature for 1 h. Subsequently, cells were incubated with γH2AX antibody, Fluor488-conjugated antibody, and DAPI. Finally, the cells were observed by Inverted Biological Microscope (Optec, BDS400, China) to observe the DNA damage level.

Secondly, we used mitochondrial transcription factor A (TFAM) and MitoTracker co-staining to detect mitochondrial DNA damage. 4T1 cells were treated with various groups: (1) Control, (2) Laser, (3) GZn NPs, (4) GZn NPs+Laser, and (5) Zn^2+^ for 2 h, respectively. The concentrations of GZn NPs were 12.5 μg/mL. The concentration of Zn^2+^ was 3 μg/mL. The group of Laser and GZn NPs+Laser was irradiated by NIR-II laser lasting 10 min. Next, cells were incubated in an incubator for 2 h. Then, the cells were stained with MitoTracker and fixed with 4% paraformaldehyde. Then, cells were treated with 0.3% Triton X-100 for 10 min and sealed with 5% BSA at room temperature for 1 h. Subsequently, cells were incubated with TFAM antibody, Fluor488-conjugated antibody, and DAPI. Finally, the cells were observed by CLSM (Olympus, FV3000RS, Japan) to observe leaked mitochondrial DNA.

**Western blot assay**

4T1 cells were treated with various groups: (1) Control, (2) Laser, (3) GZn NPs, (4) GZn NPs+Laser, and (5) Zn^2+^ for 2 h, respectively. The concentrations of GZn NPs were 12.5 μg/mL of Au. The concentration of Zn^2+^ was 3 μg/mL. The group of Laser and GZn NPs+Laser was irradiated by NIR-II laser lasting 10 min. Next, cells were incubated in an incubator for 2 h. The cells were washed thoroughly with PBS and lysed with RIPA buffer (Beyotime) together with a protease inhibitor (Servicebio). The total proteins (40 μg) were loaded onto 10% SDS-PAGE gels and transferred to 0.45 μm PVDF membranes after incubating with primary antibodies, including cGAS (1:1000), p-STING (1:1000), STING (1:1000), p-TBK1 (1:1000), TBK1 (1:1000), Actin (1:8000), and Tubulin (1:8000) at 4℃ overnight. Then, the secondary antibodies were incubated according to the manufacturer’s protocols. The dilution ratio of the antibody is recommended in accordance with the instructions. After washing off the secondary antibody, Western blot images were captured on a chemiluminescence imaging system (Bio-Rad, ChemiDoc, USA) using ECL (MeilunBio).

**Quantitative real-time PCR (qPCR)**

4T1 cells were treated with various groups: (1) Control, (2) Laser, (3) GZn NPs, (4) GZn NPs+Laser, and (5) Zn^2+^ for 2 h, respectively. The concentrations of GZn NPs were 12.5 μg/mL of Au. The concentration of Zn^2+^ was 3 μg/mL. The group of Laser and GZn NPs+Laser was irradiated by NIR-II laser lasting 10 min. Next, cells were incubated in an incubator for 2 h. The cells were washed thoroughly with PBS. Then, according to the manufacturer’s protocol, Trizol was used for extracting RNA. Complementary DNA (cDNA) was obtained by RNA reverse transcription with a cDNA reverse transcription kit. Meanwhile, the cDNA was analyzed by qPCR with SYBR qPCR Master Mix. The primer sequences used in this research are shown in Supplementary Table 1.

**ICD Detection of immunogenic cell death**

4T1 cells were treated with various groups: (1) Control, (2) Laser, (3) GZn NPs, (4) GZn NPs+Laser, and (5) Zn^2+^ for 2 h, respectively. The concentrations of GZn NPs were 12.5 μg/mL of Au. The concentration of Zn^2+^ was 3 μg/mL. The group of Laser and GZn NPs+Laser was irradiated by NIR-II laser (1064 nm, 1.5 W/cm^2^) lasting 10 min. Next, cells were incubated in an incubator for 2 h. Then, the cells were washed three times with PBS and fixed with 4% paraformaldehyde. Then, cells were treated with 0.3% Triton X-100 for 10 min (except for CRT immunofluorescence staining) and sealed with 5% BSA at room temperature for 1 h. Subsequently, cells were incubated with CRT and HMGB1 antibody, Fluor488-conjugated antibody, and DAPI. Finally, the cells were observed by Inverted Biological Microscope (Optec, BDS400, China). The release of HMGB1 and ATP was detected by the corresponding Assay Kit according to the manufacture’s protocols.

**Animal experiments**

BALB/c female mice (3-5 weeks, 13-15 g) were purchased from Shanghai Jihui Laboratory Animal Care Co., Ltd. All mice were fed in a specific pathogen-free, light-cycled, and temperature-controlled standard barrier environment. All animal experiments were conducted in a standard barrier environment of the Laboratory Animal Center of Hangzhou Normal University (Hangzhou, China). All animal experiments were performed under the regulations of Laboratory animal management and the approval of the Ethics Committee of Hangzhou Normal University (20250930-02).

**In vivo Anti-Tumor efficiency of GZn NPs**

4T1 cells (1×10⁶ cells per mouse) were injected into the left mammary fat pads of female BALB/c mice. When the tumor volume reached approximately 50-70 mm³, the tumor-bearing mice were randomly divided into five groups (n=8). Each group was treated with (1) PBS, (2) Zn^2+^, (3) GZn NPs, (4) Laser, (5) GZn NPs+Laser. The mice in different groups were injected with PBS, Zn^2+^ (4mg/kg of Zn) and GZn NPs (4mg/kg of Au) on days 6, 9, and 12, respectively. Additionally, group Laser and GZn NPs+Laser were treated with the 1064 nm laser on days 10 and 15 (1064 nm, 2.0 W/cm², 5 min). During the treatment period, tumor volume and the mice body weight were measured every two days. On day 18, all the mice were sacrificed and the tumors were excised, weighed, photographed, and preserved for further analysis.

The collected tumor tissues were lysed with RIPA lysis buffer (Beyotime) in the presence of protease and phosphatase inhibitors (Servicebio), followed by homogenization and centrifugation. The obtained protein samples were subjected to WB analysis. The levels of IFNβ in the tumor samples were quantified using the Mouse IFNβ ELISA kit (MULTI SCIENCES).

To evaluate the activation of innate immunity, tumor-draining lymph nodes (TDLNs) were collected for DC analysis on day 11. The TDLNs were ground, filtered, and centrifuged to obtain single cell suspensions. DCs were stained with anti-CD45-BV605 (BioLegend), anti-CD11c-FITC (BioLegend), anti-CD86-APC (BioLegend), and anti-CD80-PE (BioLegend) for Flow cytometry analysis.

**Tumor rechallenge studies**

Firstly, the 4T1 cells (1×10⁶ cells per mouse) were injected into the left mammary fat pads of female BALB/c mice to construct the primary tumor model. When the tumor volume reached approximately 50-70 mm^3^, the 4T1 tumor-bearing mice were randomly divided into five groups (n=5). Each group was treated with (1) PBS, (2) Zn^2+^, (3) GZn NPs, (4) Laser, (5) GZn NPs+Laser. The mice in different groups were injected with PBS, Zn^2+^ and GZn NPs on days 6 and 8, respectively. Additionally, group Laser and GZn NPs+Laser were treated with the 1064 nm laser after 24h post-injection. On day 21, the primary tumors were removed by surgery. On day 30, these mice were rechallenged with 4T1 cells (5×10^6^) on the other side to construct a rechallenged tumor model. After that, the tumor volume was measured for 18 days.

To evaluate the activation of adaptive immunity, the spleens and rechallenged tumors were collected for effector T cells in the tumor and memory T cells in the spleen on day 48. The rechallenged tumors were enzymatically digested to produce single-cell suspensions. Effector T cells were stained with anti-CD45-BV605 (BioLegend), anti-CD3-APC (BioLegend), anti-CD4-FITC (BioLegend), and anti-CD8a-PC7 (BioLegend) for Flow cytometry analysis. Memory T cells were stained with anti-CD45-BV605 (BioLegend), anti-CD3-APC (BioLegend), anti-CD44-PE (BioLegend), and anti-CD62L-PB450 (BioLegend) for flow cytometry analysis.

**Biosafety assay in vivo**

Healthy BALB/c female mice were randomly divided into three groups (n=5) and injected with 100 μL of PBS (Control), 50 μg/mL GZn NPs, or 100 μg/mL GZn NPs (0, 3 and 6 days). Body weight was monitored closely throughout the experiment. After 14 days, all mice were sacrificed at predetermined time points. The blood samples were collected for routine blood examination and blood biochemistry analyses. The major organs (heart, liver, spleen, lung, and kidney) of the mice were harvested and stained with H&E for histological analysis.

**Statistical analysis**

Data are expressed as the mean ± SD. Statistical significance was assessed by one-way ANOVA with Tukey's post-hoc test. Data analysis was performed with GraphPad Prism 9.0 and Origin 2022. (*p < 0.05, **p < 0.01, ***p < 0.001, ****p < 0.0001).

**Supplementary Figures**


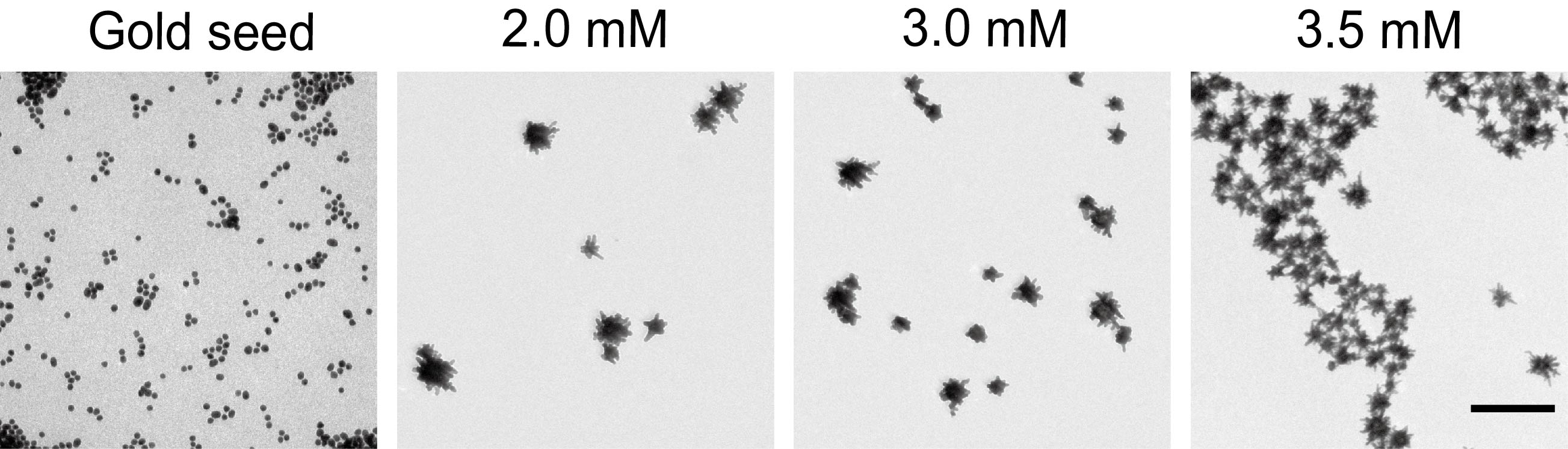


**Figure S1.** TEM images of gold seed and GNS synthesized with different concentrations of AgNO_3_. Scale bar: 100 nm.


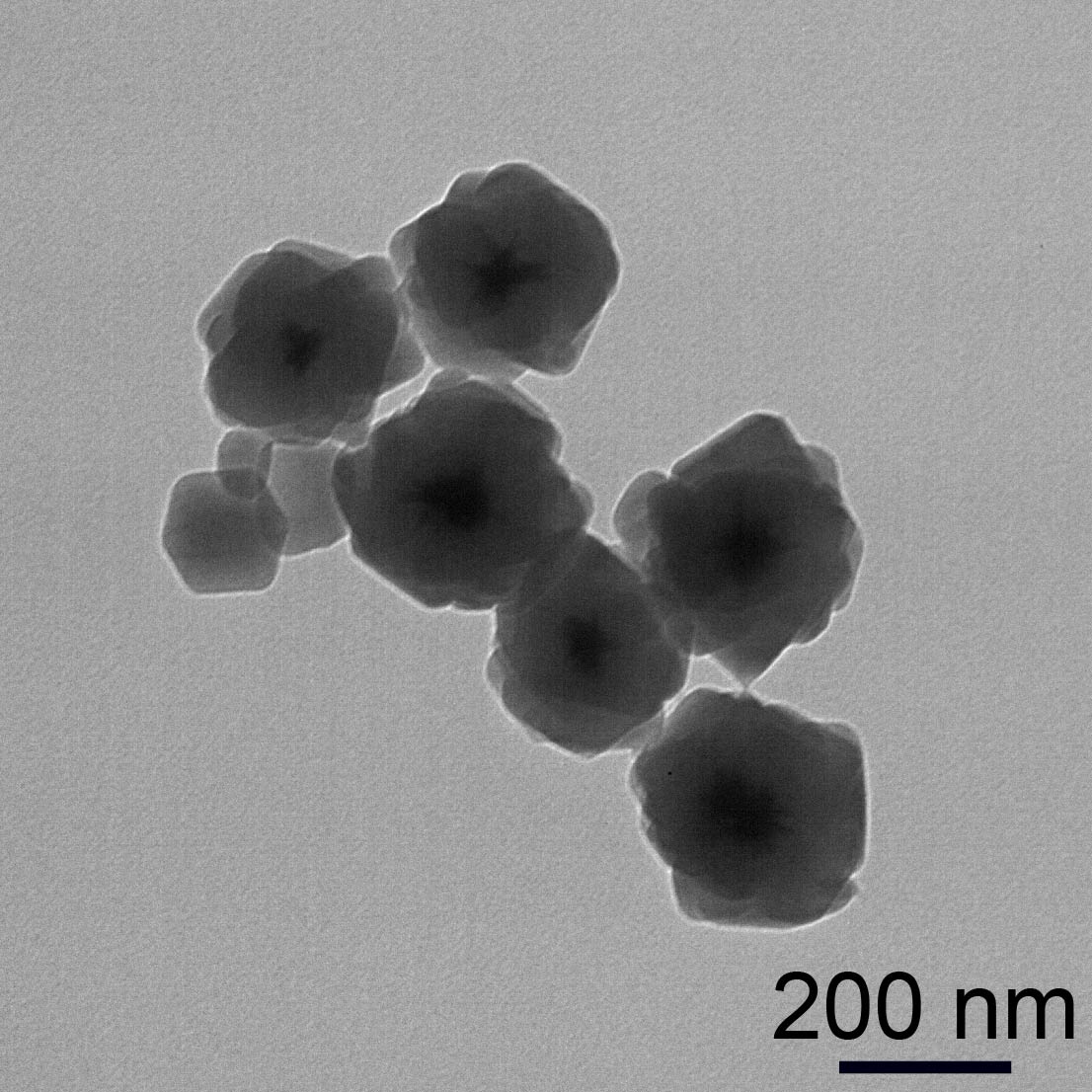


**Figure S2.** TEM images of GZn NPs.


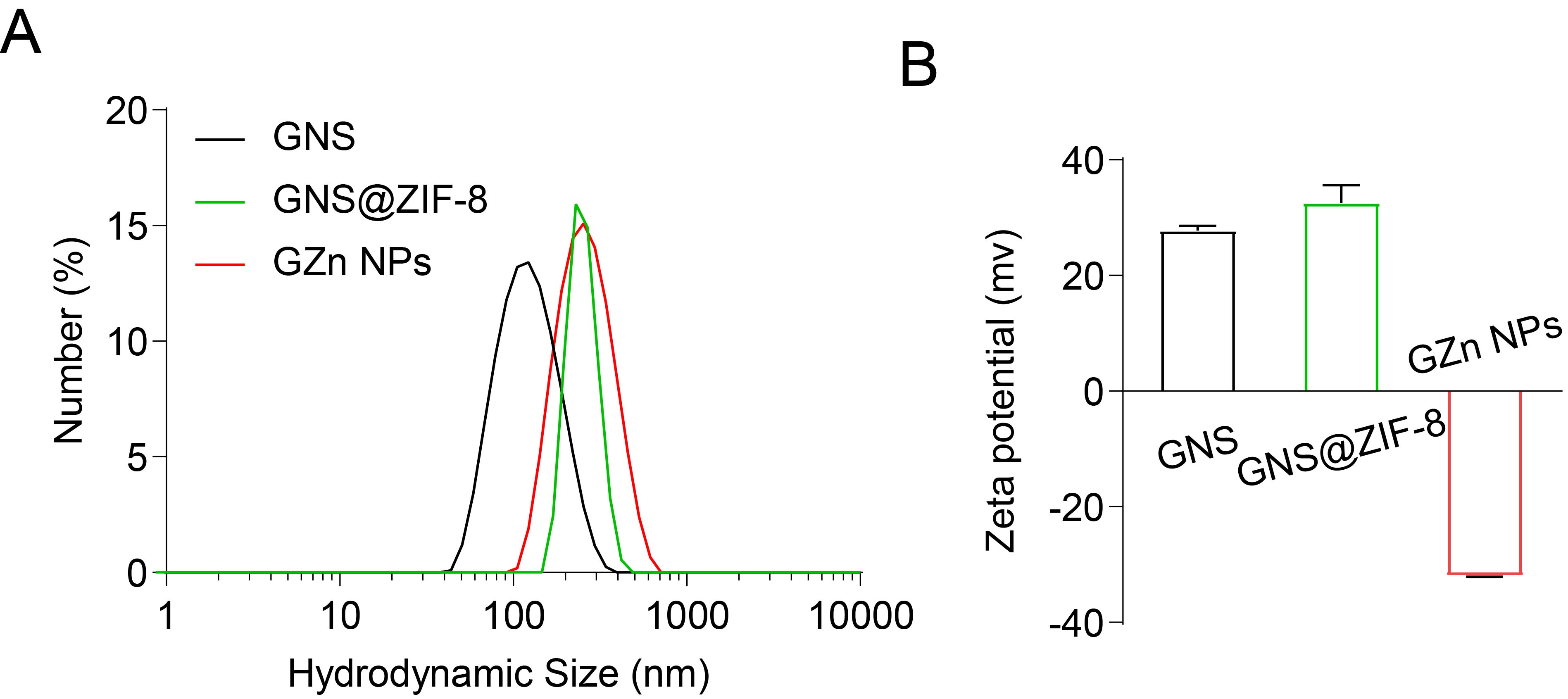


**Figure S3.** (A) Dynamic light scattering and (B) Zeta potential analysis. Data are presented as mean ± S.D. (n=3).


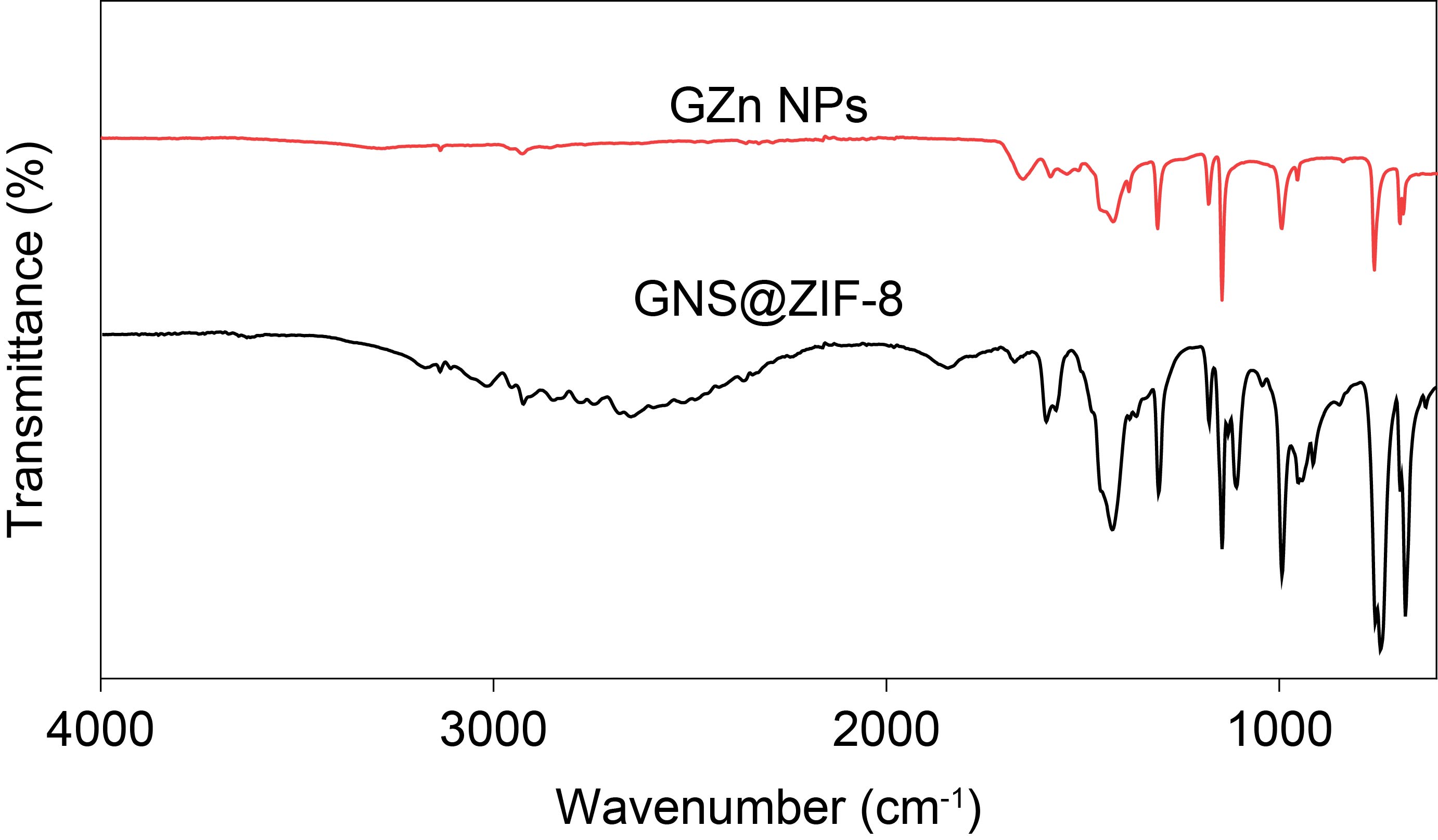


**Figure S4.** Fourier transform infrared (FTIR) spectra of GNS@ZIF-8 and GZn NPs.


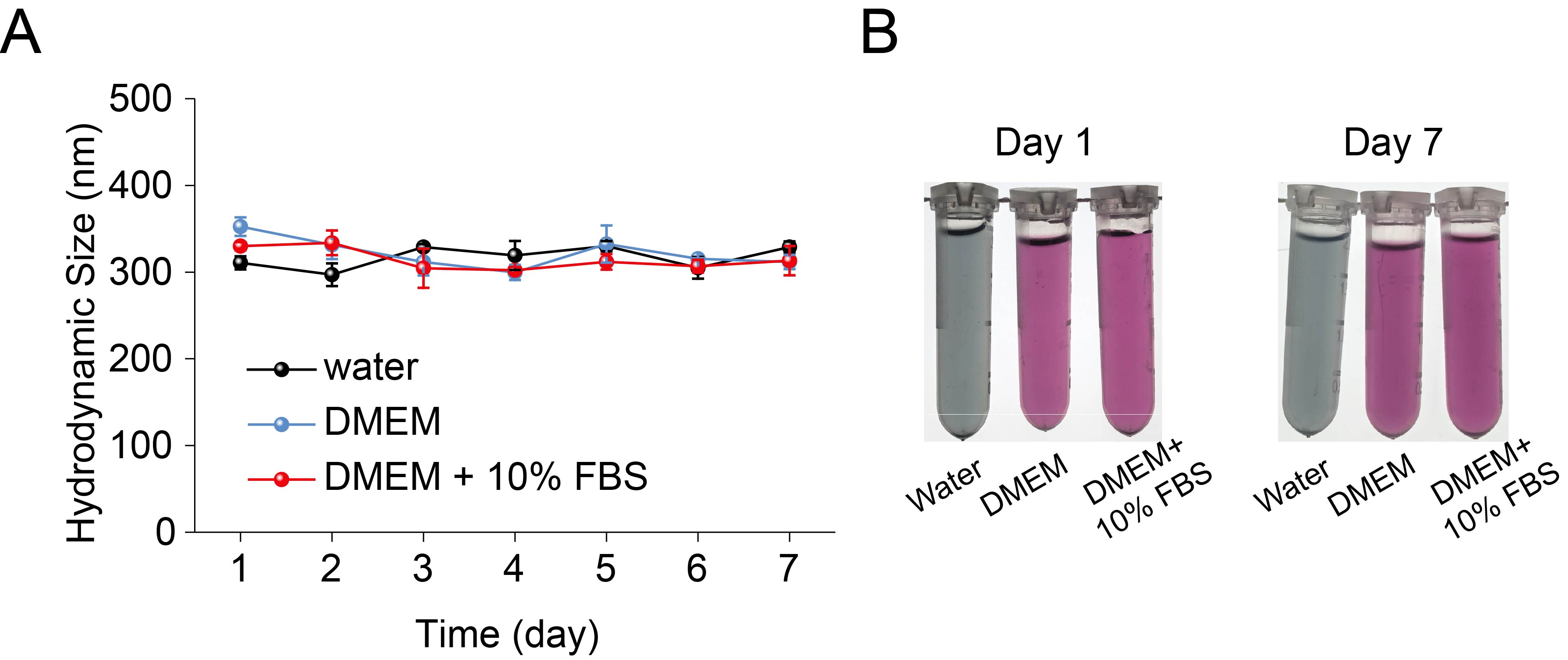


**Figure S5.** (A) Hydrodynamic size changes of GZn NPs in different solutions (Water, DMEM, and DMEM+10% FBS) for 7 days. (B) Corresponding photographs of the seventh day. Data are presented as mean ± S.D. (n=3).


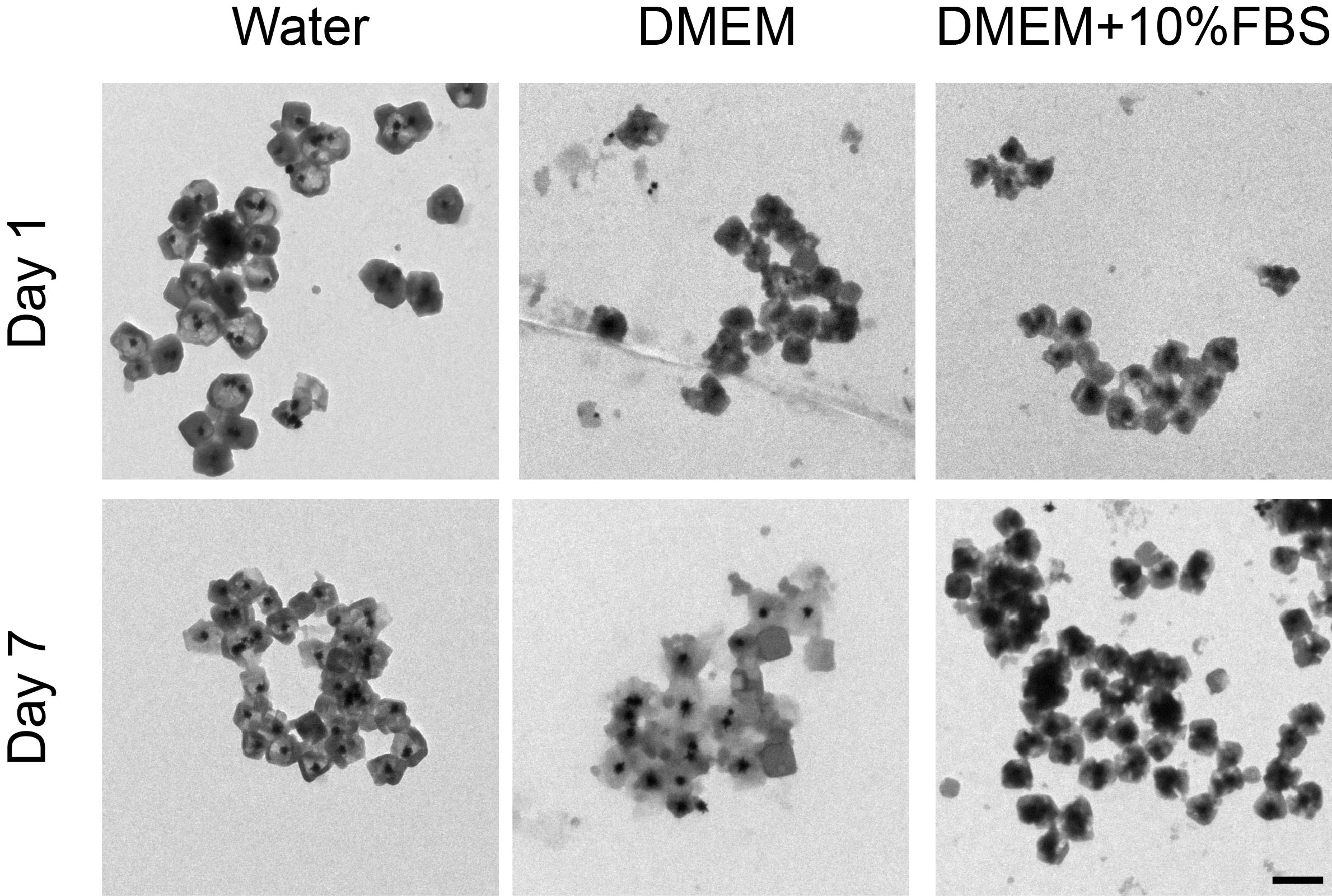


**Figure S6.** TEM images of GZn NPs in different solutions (Water, DMEM, and DMEM+10% FBS) for 7 days. Scale bar: 500 nm.


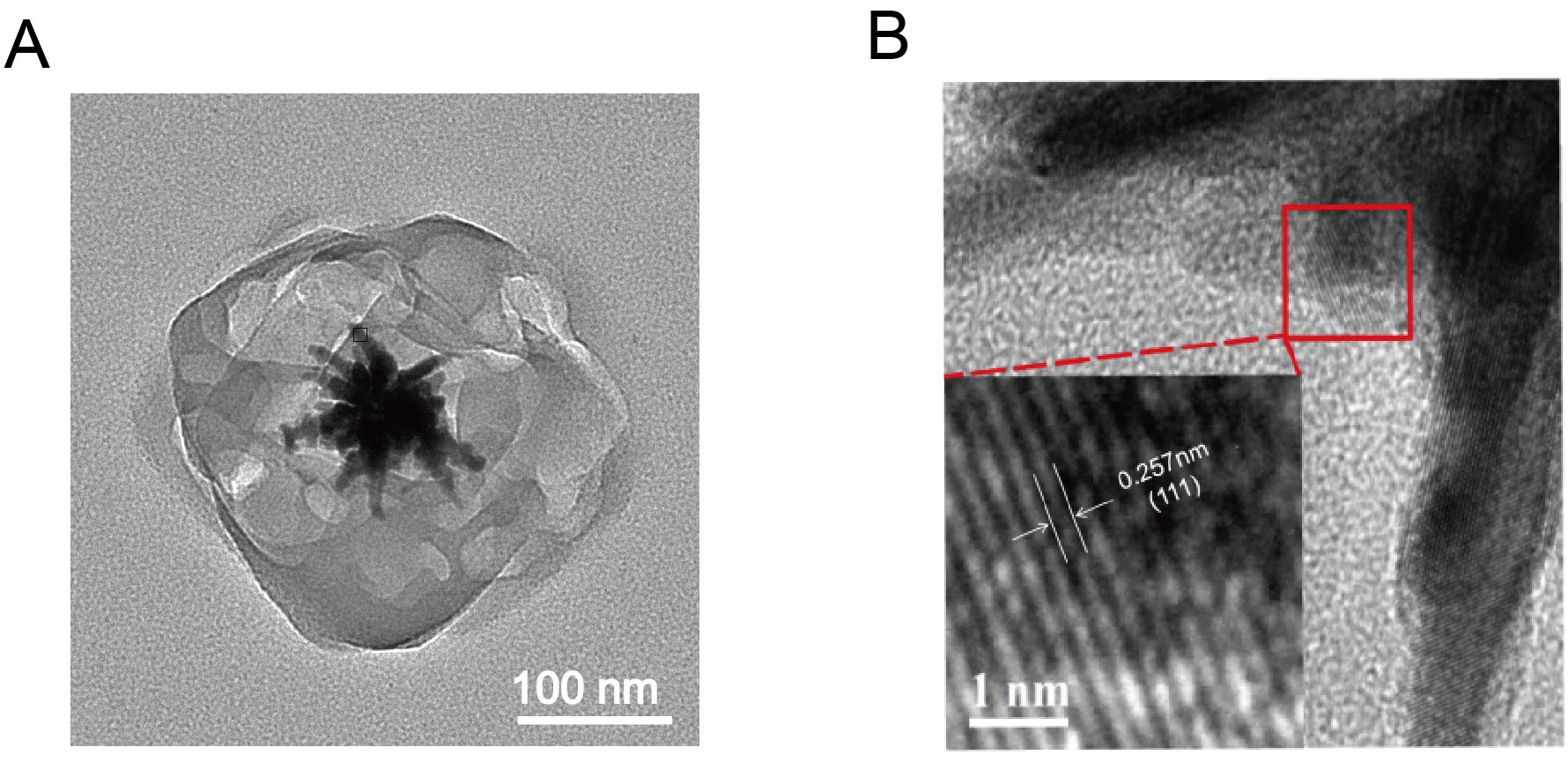


**Figure S7.** (A) High-resolution transmission electron microscopy (HRTEM) of GZn NPs. (B) Magnifying TEM images of GZn NPs.


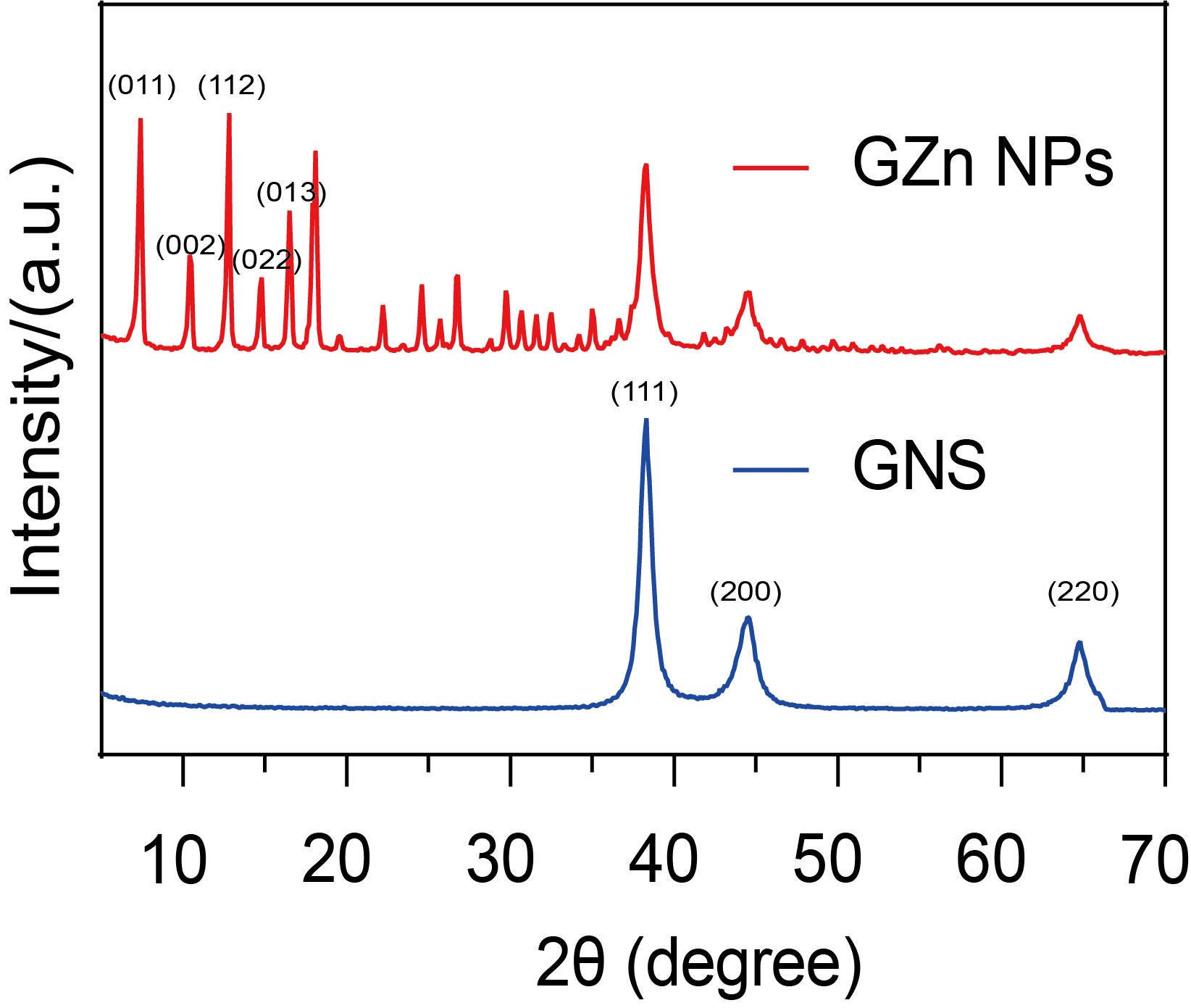


**Figure S8.** XRD patterns of GNS and GZn NPs.


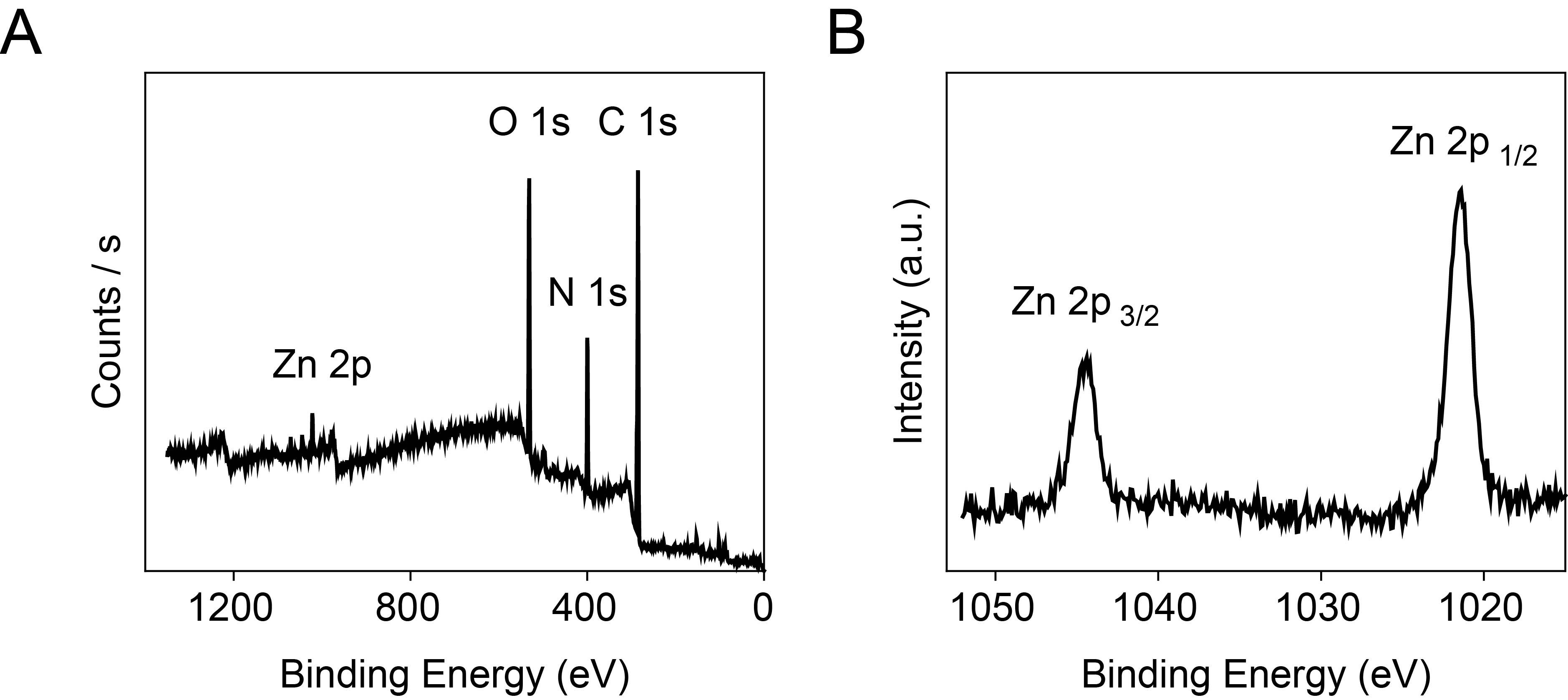


**Figure S9.** (A) XPS patterns of GZn NPs. (B) High-resolution XPS results of Zn 2p peaks in GZn NPs.


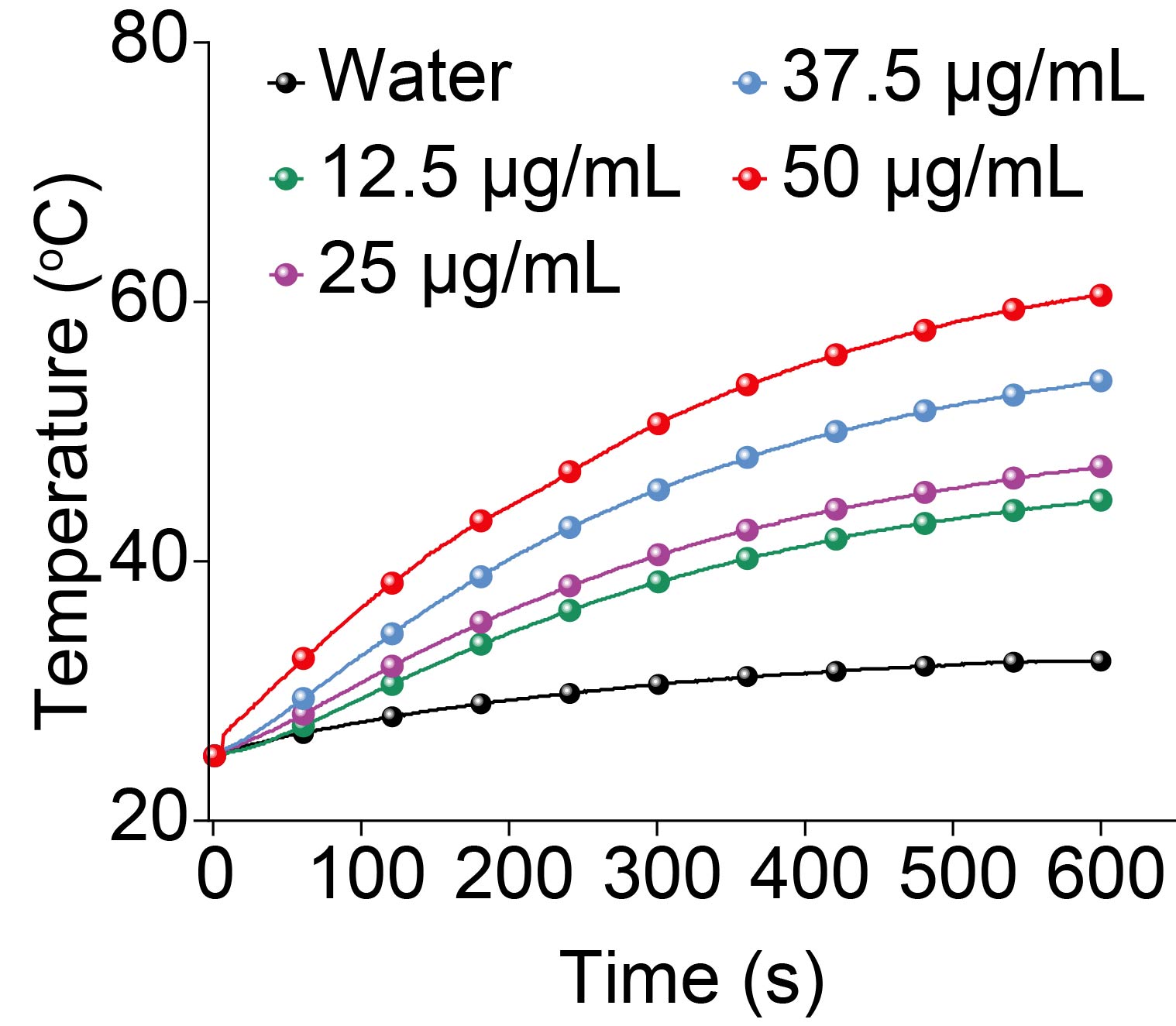


**Figure S10.** Temperature curves of GNS with different concentrations after 1064 nm laser irradiation (0-50 μg/mL of Au, 2.0 W/cm^2^).


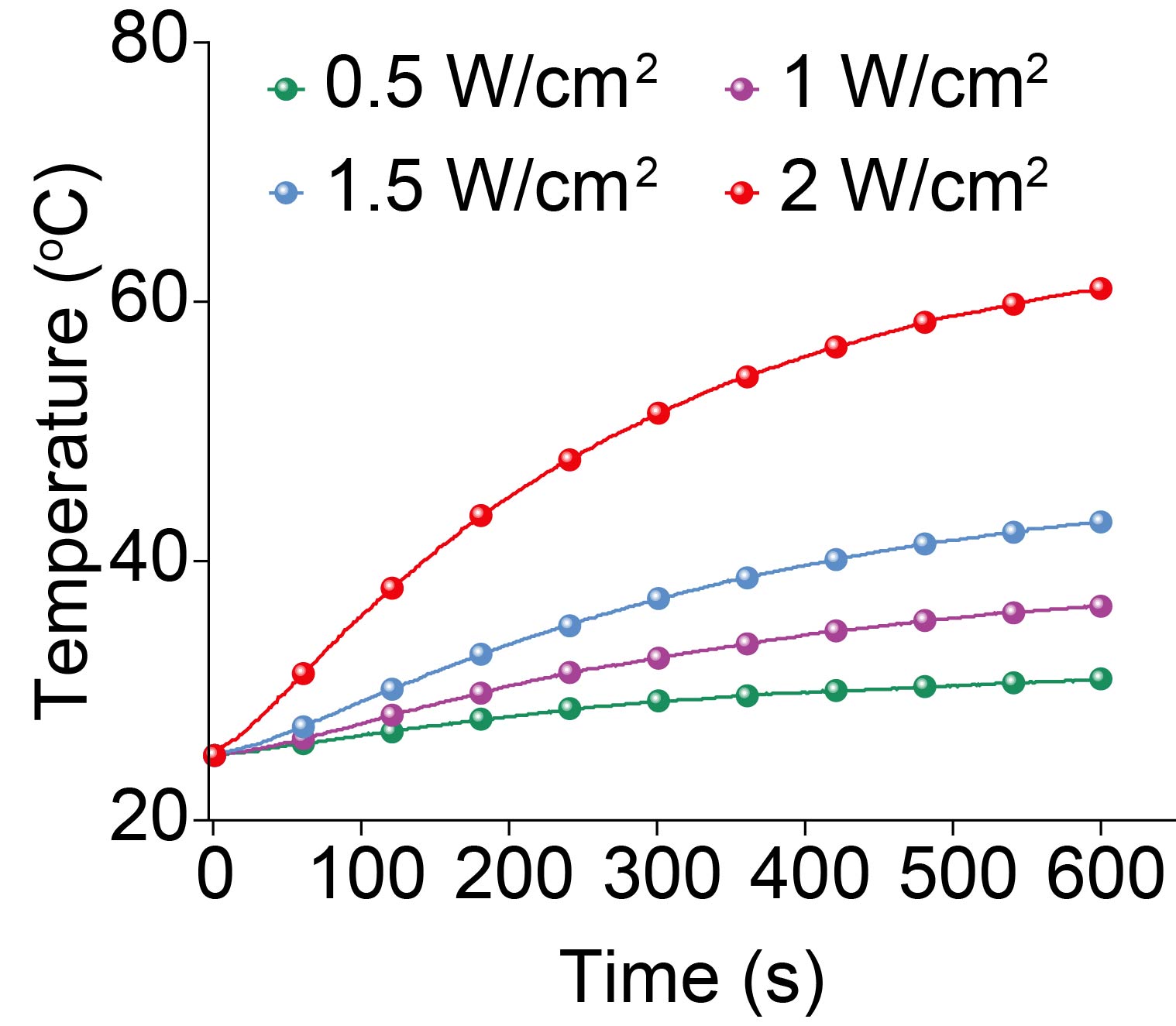


**Figure S11.** Temperature curves of GNS with various laser powers after 1064 nm laser irradiation (50 μg/mL of Au, 0.5-2.0 W/cm^2^).


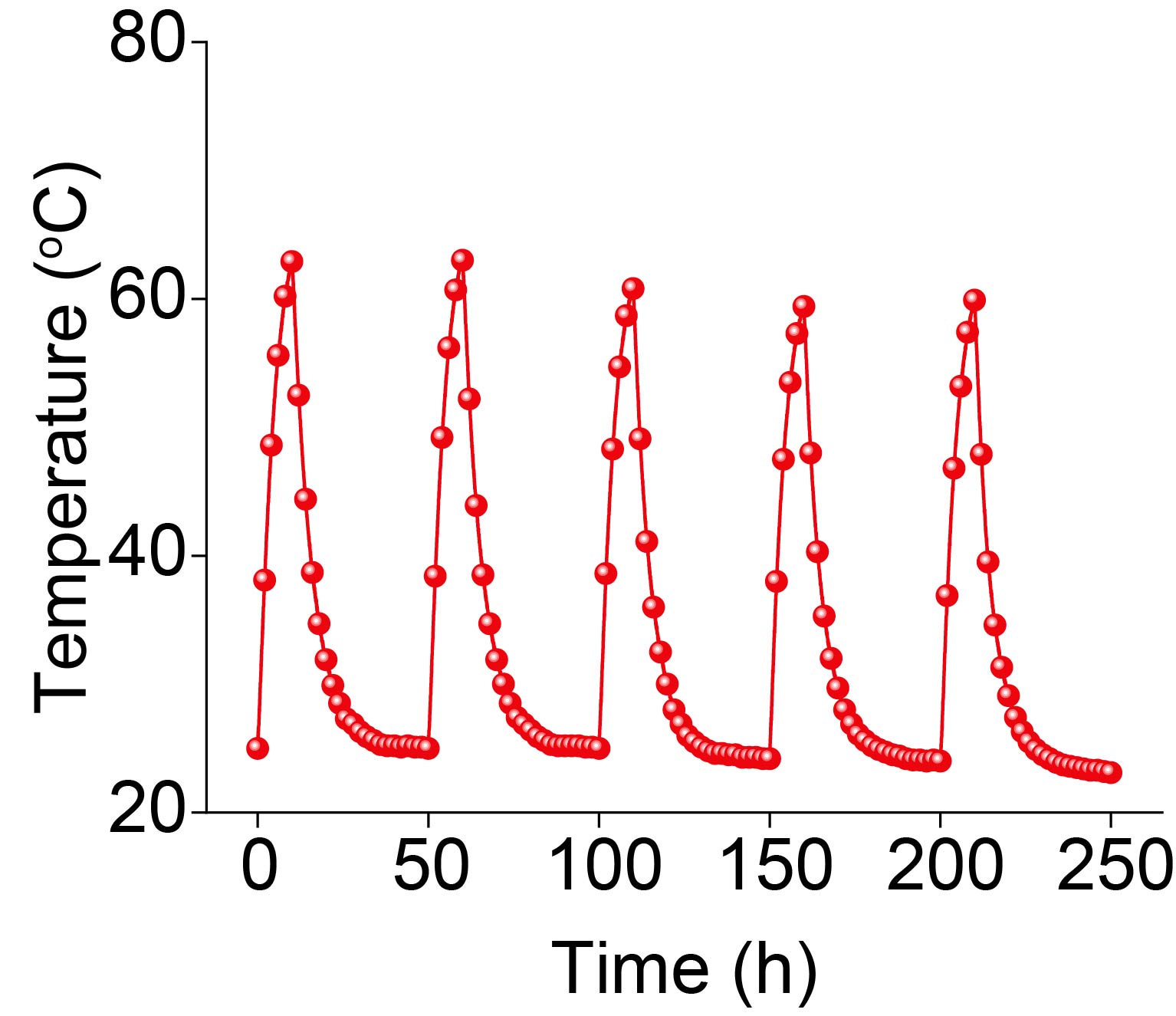


**Figure S12.** Photothermal stability of GNS (50 μg/mL of Au) after five cycles of laser on/off (10 min for each irradiation).


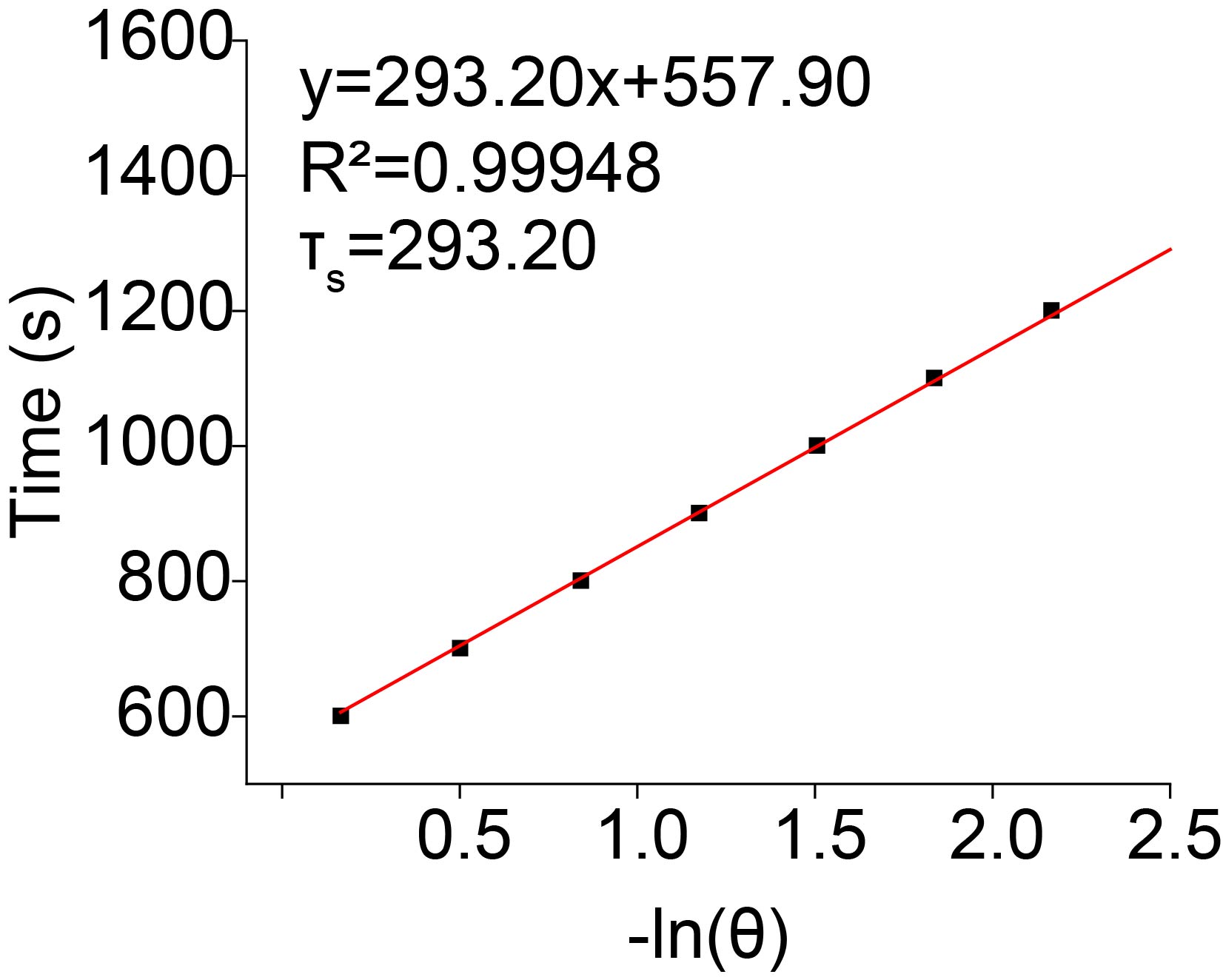


**Figure S13.** Linear regression data acquired from the cooling period.


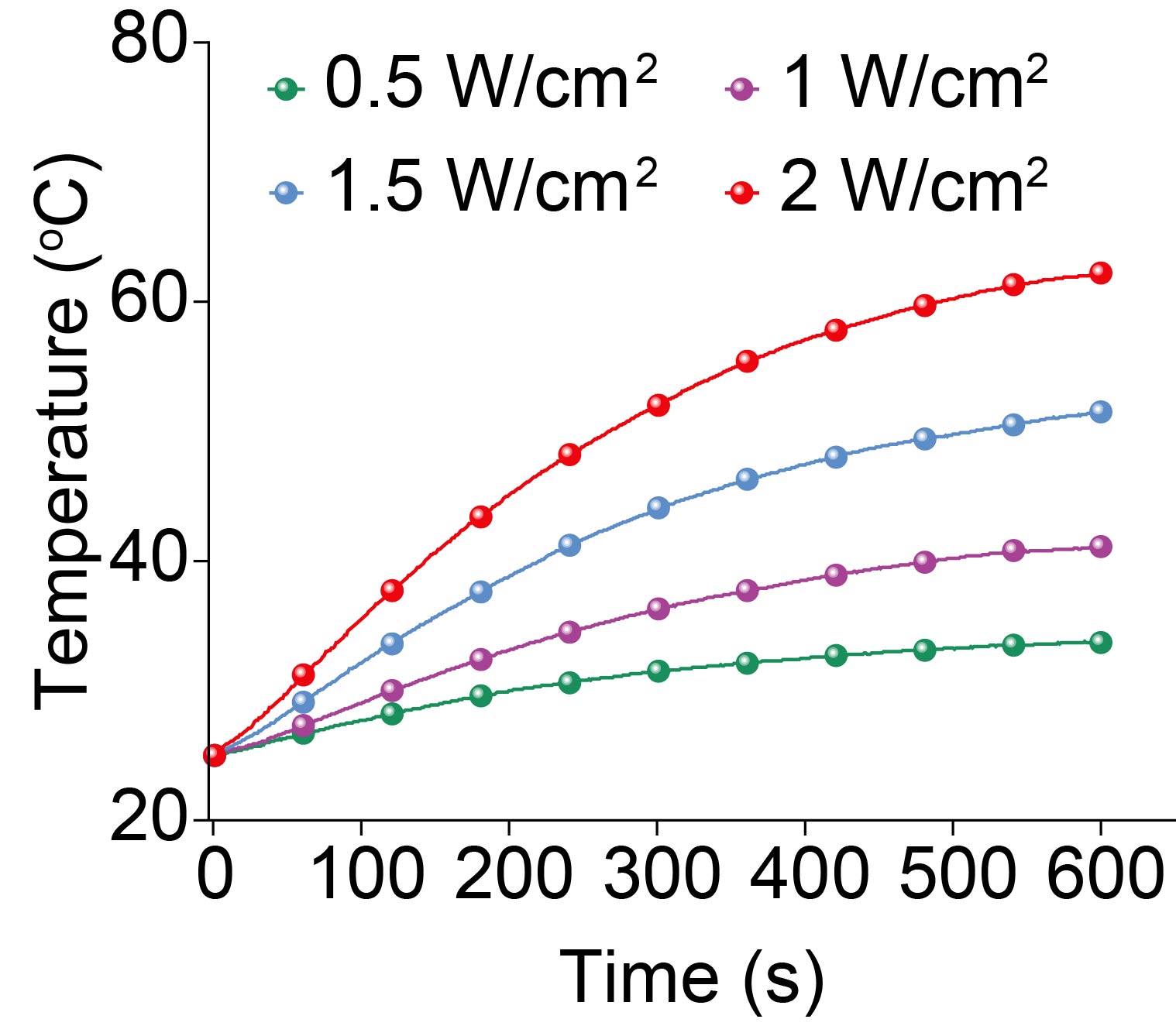


**Figure S14.** Temperature curves of GZn NPs with various laser powers after 1064 nm laser irradiation (50 μg/mL of Au, 0.5-2.0 W/cm^2^).

**
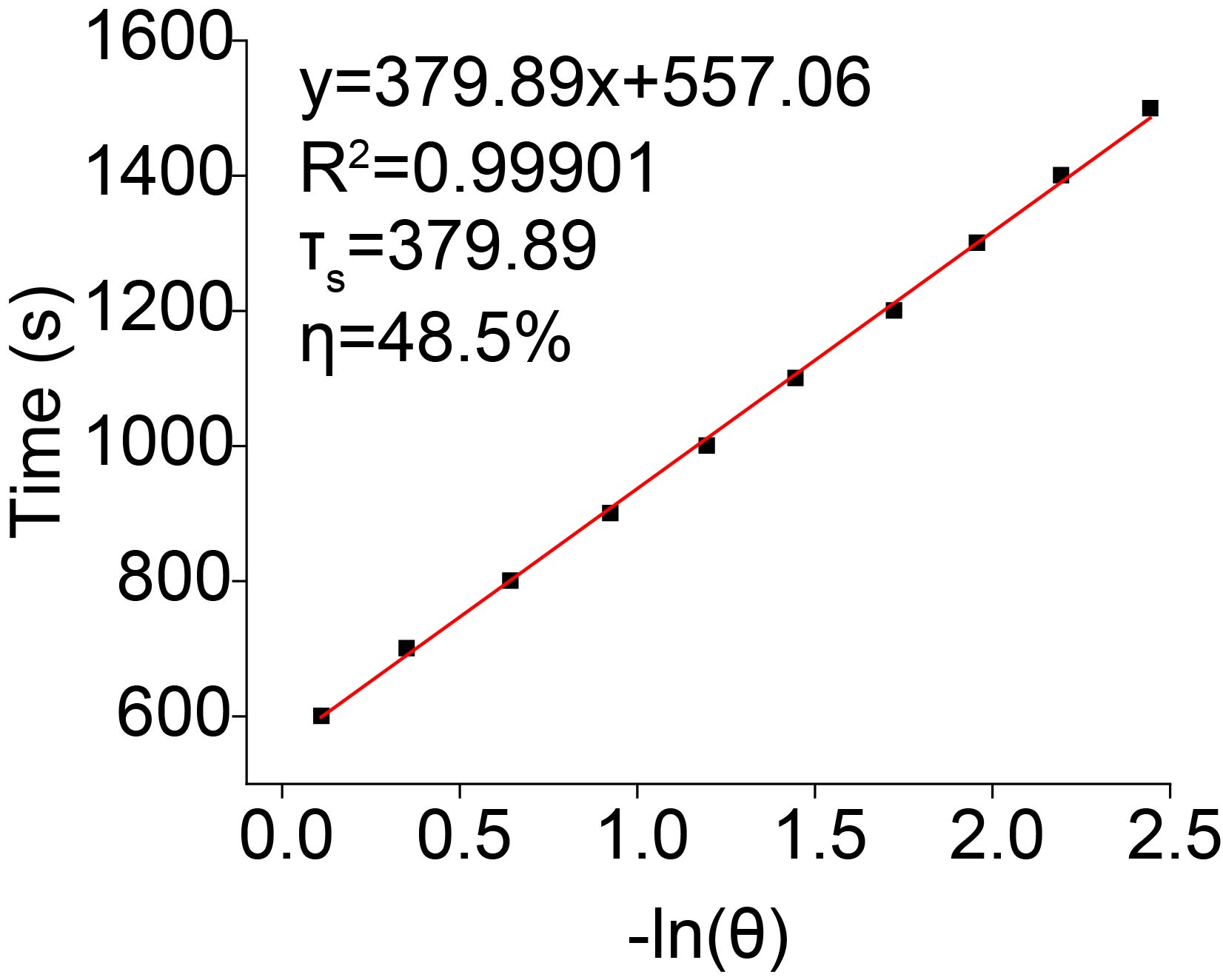
**

**Figure S15.** Linear regression data acquired from the cooling period.


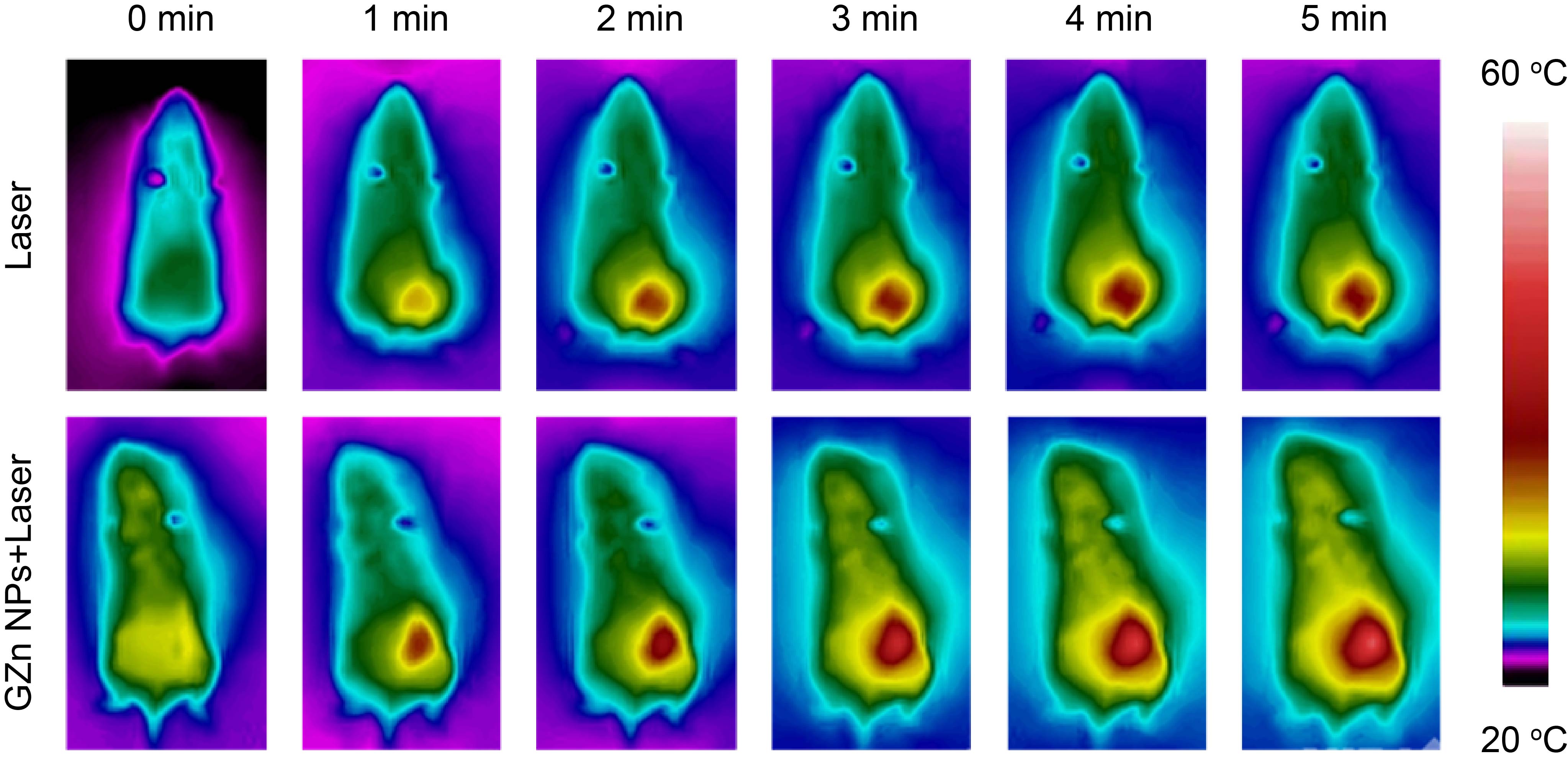


**Figure S16.** In vivo infrared thermal images of the tumor sites in 4T1-bearing BALB/c mice in different groups (PBS and GZn NPs) after laser irradiation for 0-5 min. (1064 nm, 2 W/cm^2^).


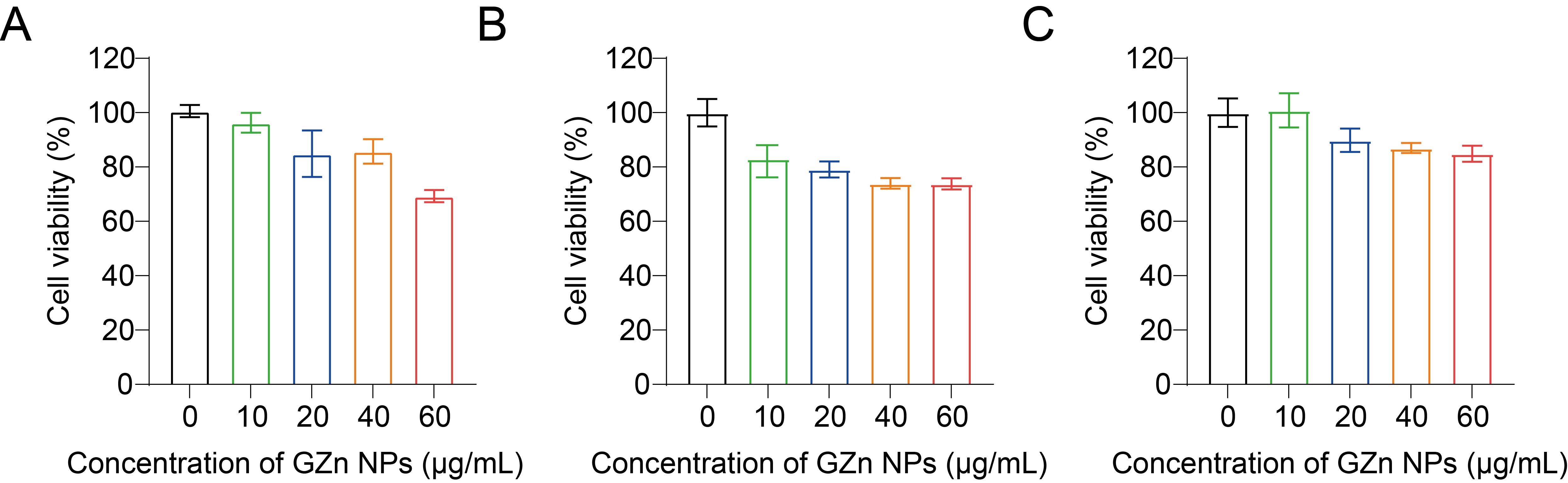


**Figure S17.** Cell viability of 4T1 cells, 3T3 cells, and HUVEC cells treated with GZn NPs for 24 h at different concentrations. Data are presented as mean ± S.D. (n=3).


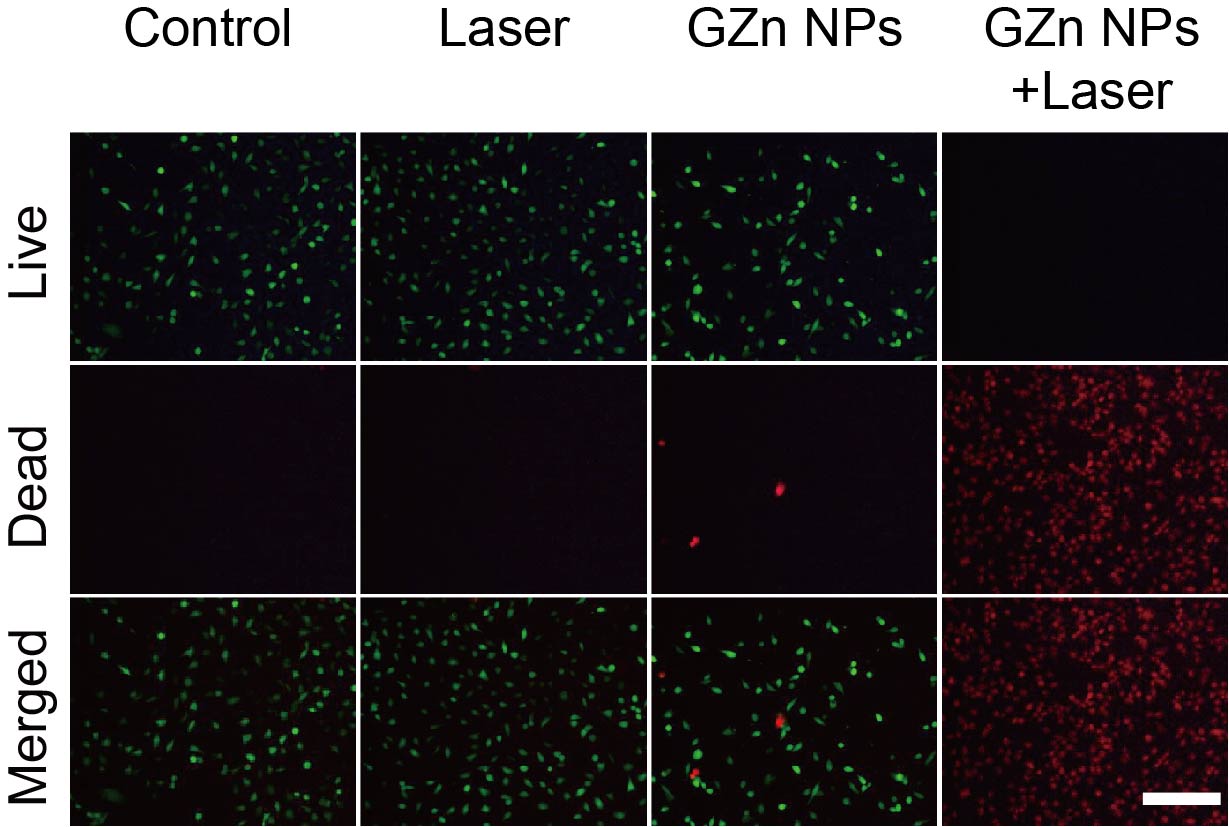


**Figure S18.** Live/dead cell staining of corresponding groups. Scale bar: 100 μm.


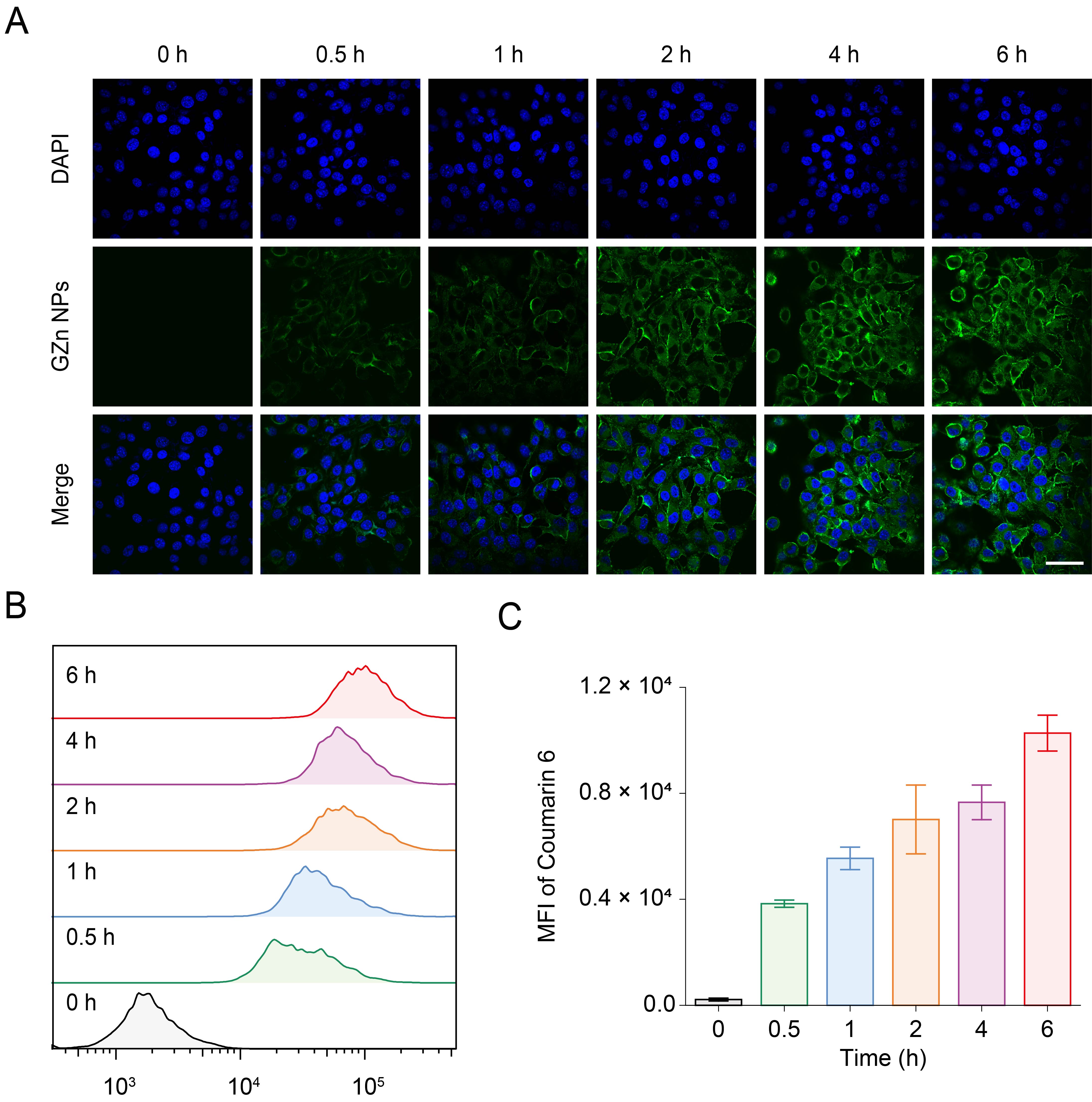


**Figure S19.** Cellular uptake of GZn NPs. (A) CLSM images of 4Tl cells treated with coumarin 6-labeled GZn NPs for 0.5, 1, 2, 4, and 6 h. The GZn NPs are stained by coumarin 6 with green fluorescence, and cell nuclei are stained by DAPI with blue fluorescence. Scale bar: 50 μm. (B-C) FCM of 4T1 cells coincubated with coumarin 6-labeled GZn NPs for 0.5, 1, 2, 4, and 6 h.


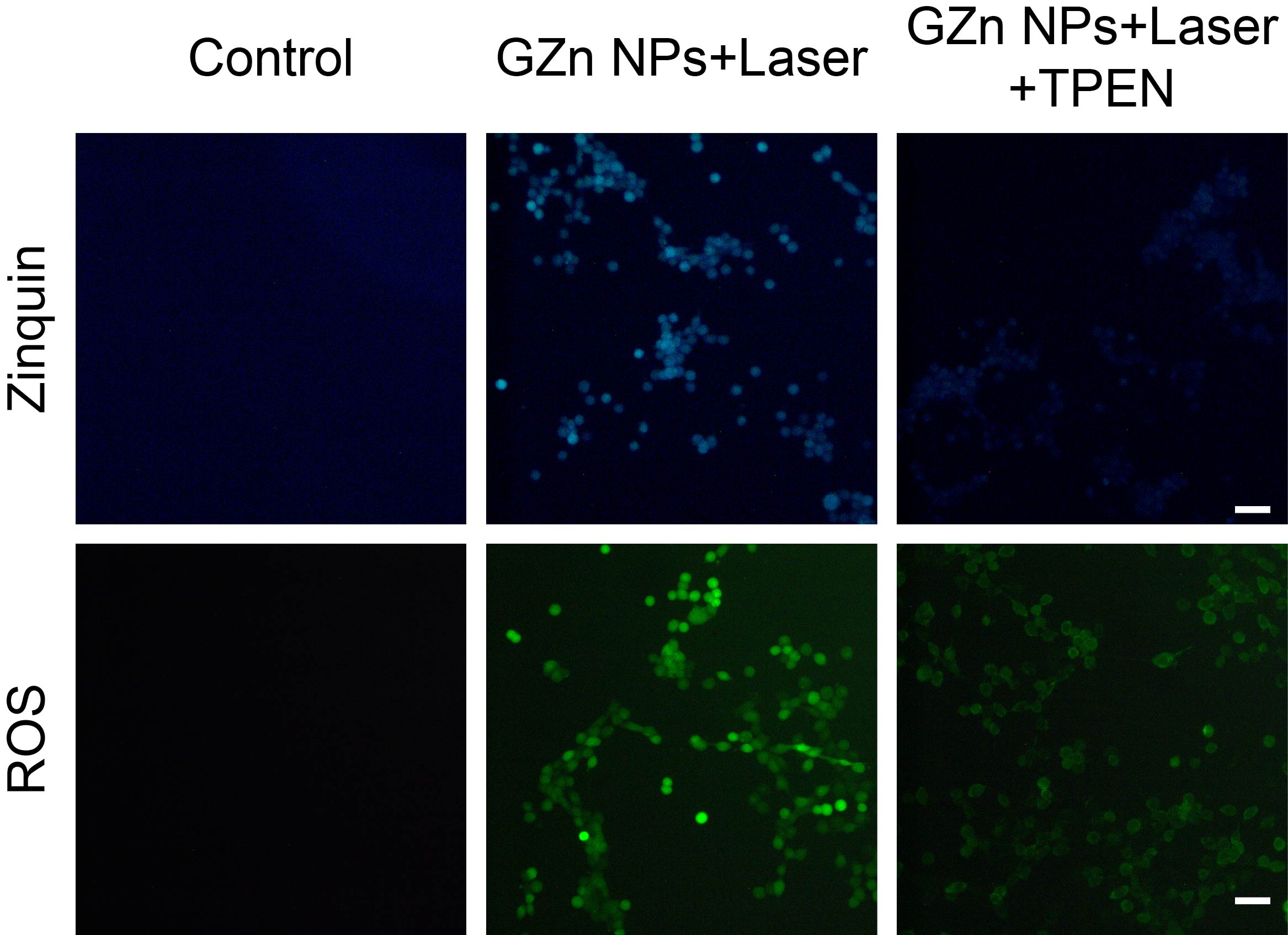


**Figure S20.** Representative images by the Zinquin and DCFH-DA staining. Scale bar: 50 μm.


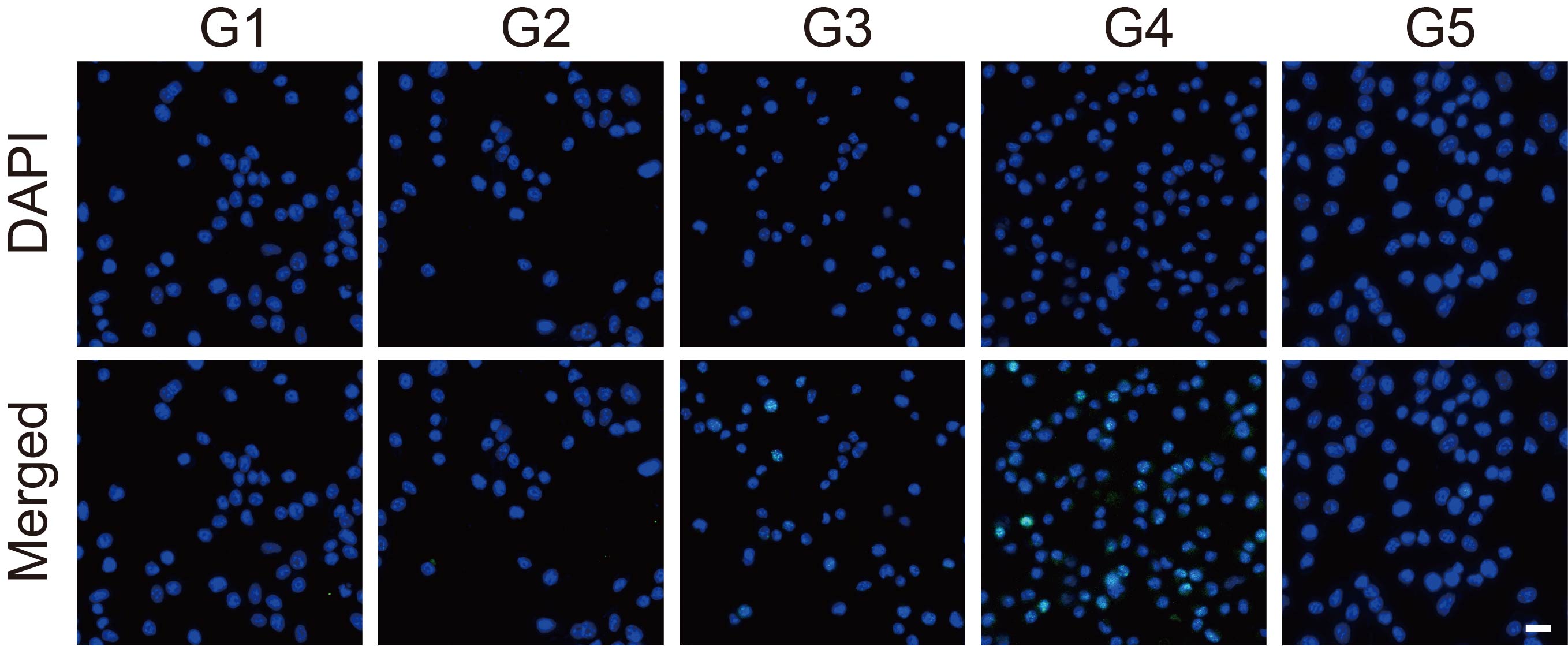


**Figure S21.** Fluorescence images of the DNA damage marker of γH2AX (G1: Control, G2: Laser, G3: GZn NPs, G4: GZn NPs+Laser, and G5: Zn^2+^). Scale bar: 20 μm.


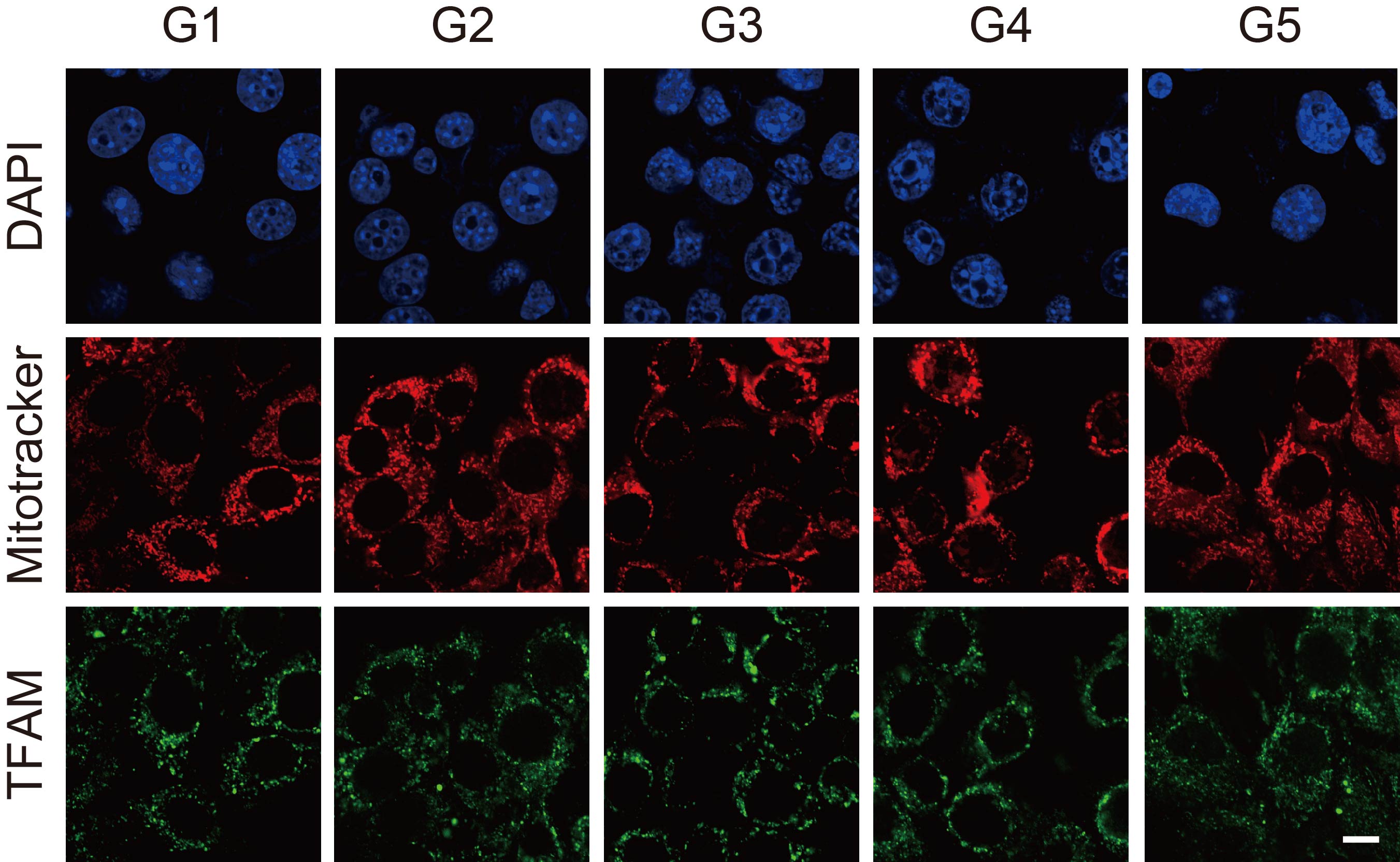


**Figure S22.** CLSM images stained with TFAM (mtDNA, green), mitotracker (mitochondrial, red) and DAPI (nucleus, blue). (G1: Control, G2: Laser, G3: GZn NPs, G4: GZn NPs + Laser, and G5: Zn^2+^). Scale bar: 10 μm.


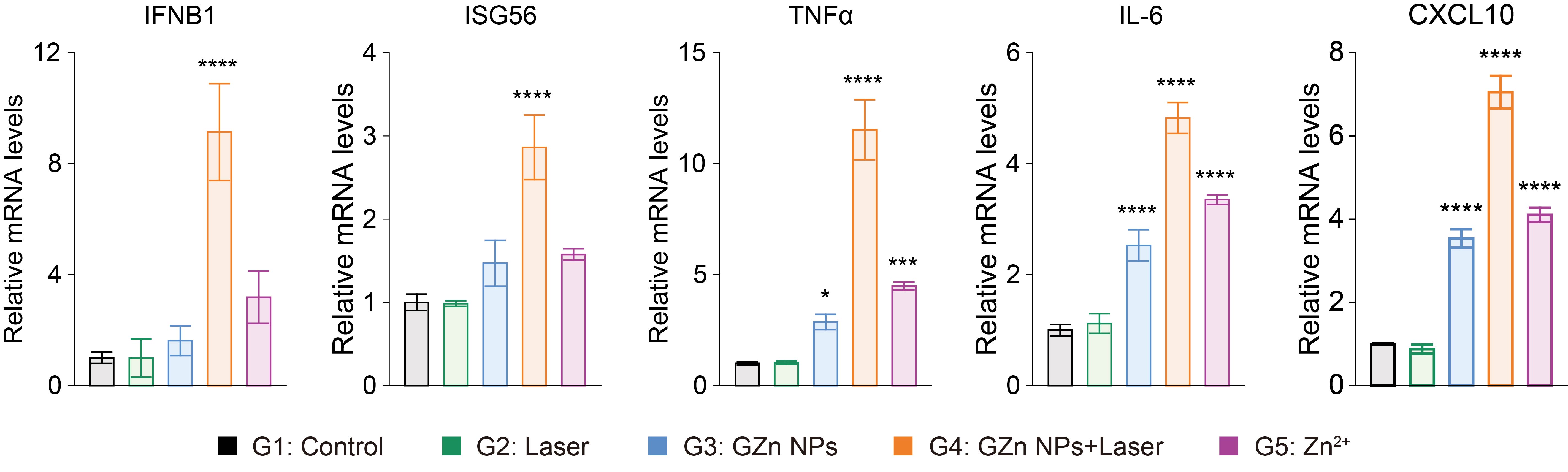


**Figure S23.** The expression of IFNB1, ISG56, TNFα, IL-6, and CXCL10 in 4T1 with the indicated treatment was detected using real-time PCR (n=3).


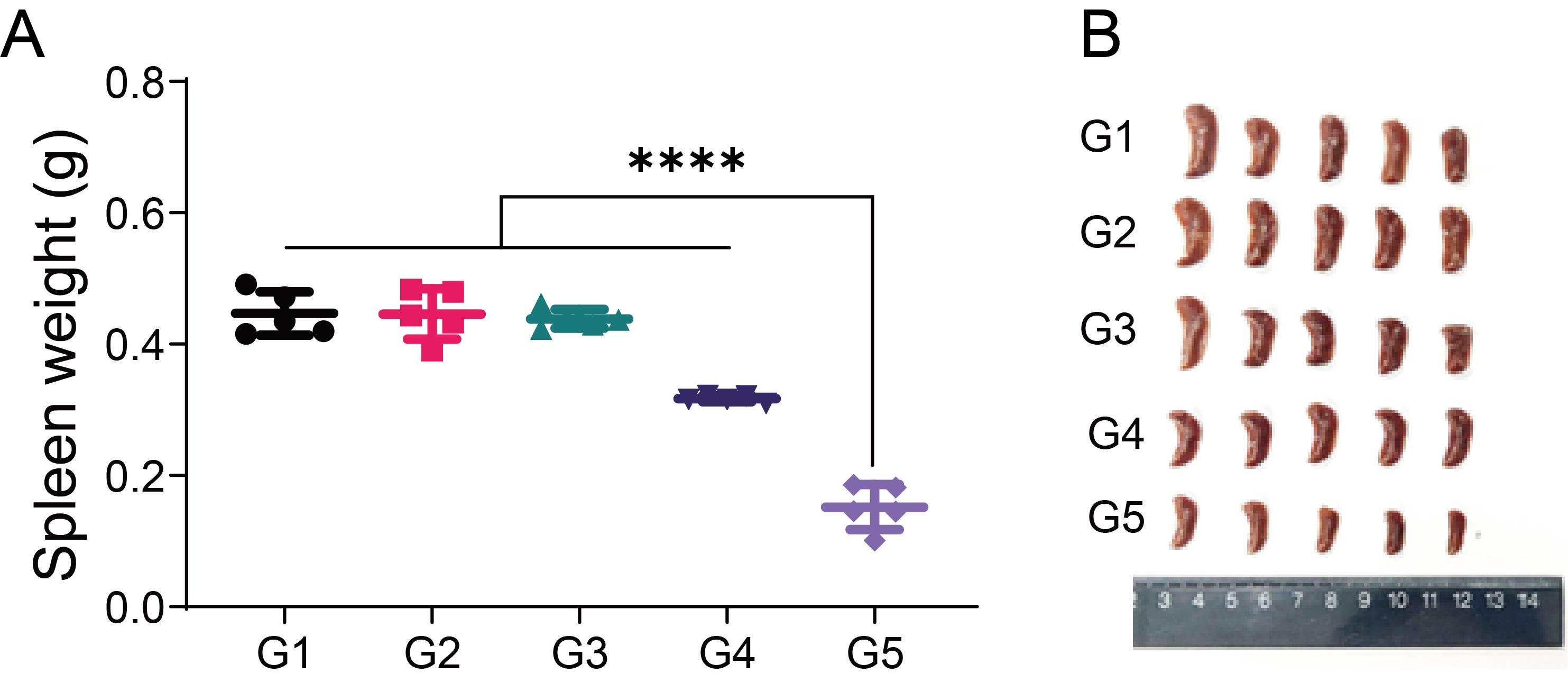


**Figure S24.** (A) Spleen weight and (B) pictures of mice after various treatments. (G1: Control, G2: Zn^2+^, G3: GZn NPs, G4: Laser, and G5: GZn NPs+Laser). Data are presented as mean ± S.D. (n=5) (*****p* < 0.0001).


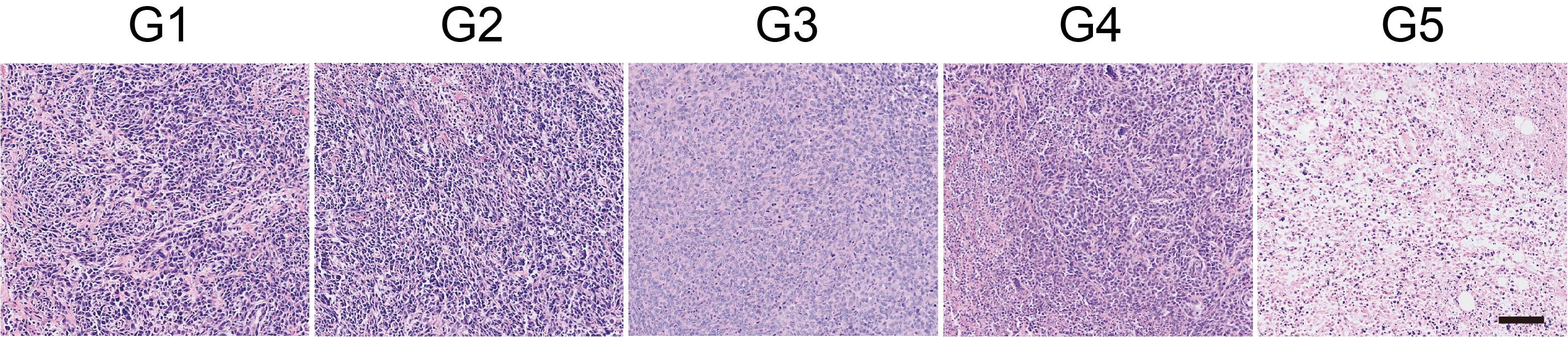


**Figure S25.** H＆E staining of tumors extracted from mice after the indicated treatments. (G1: Control, G2: Zn^2+^, G3: GZn NPs, G4: Laser, and G5: GZn NPs+Laser). Scale bar: 100 μm.


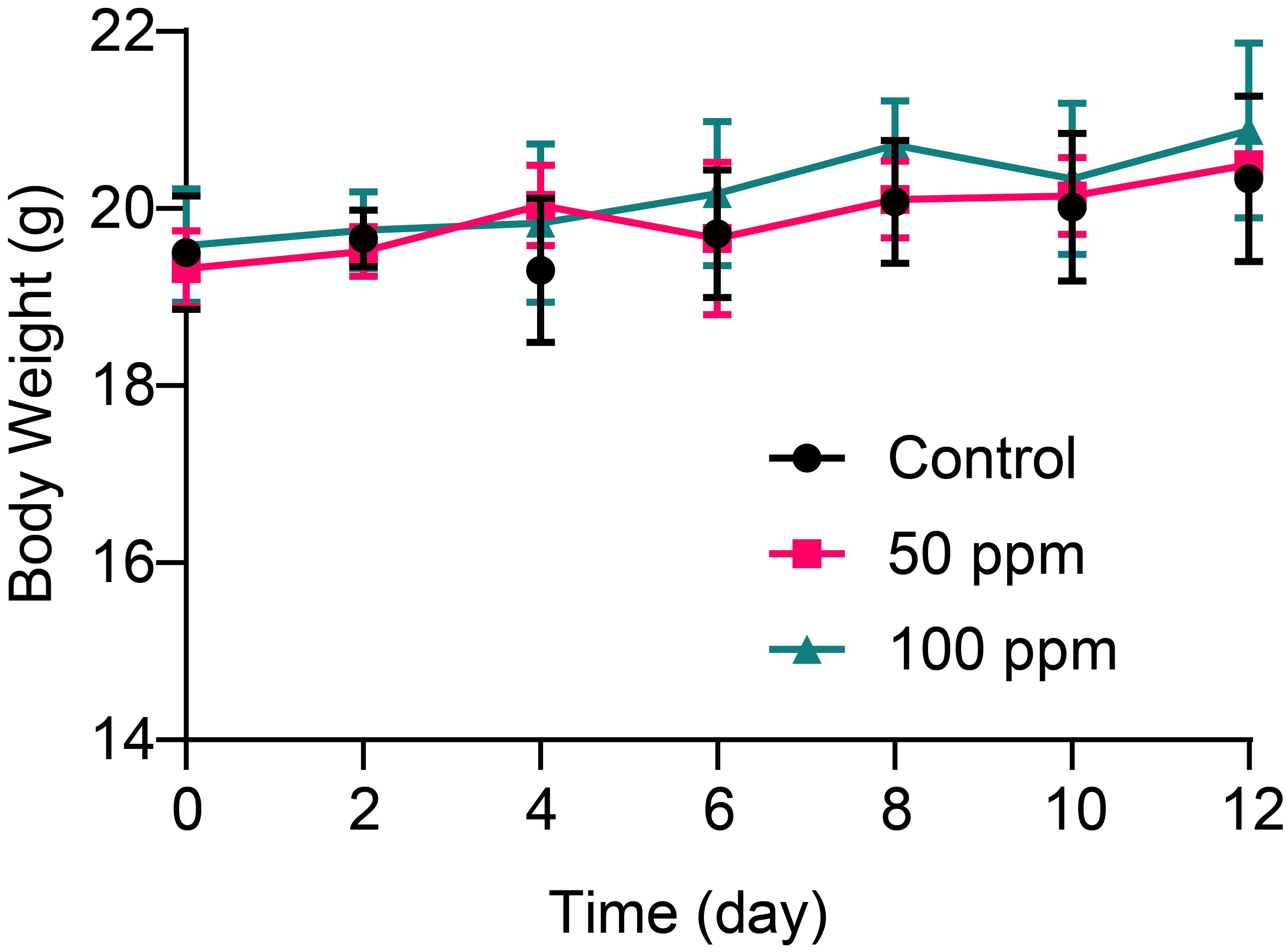


**Figure S26.** Body weight of the mice in different groups during the experiment. Data are presented as mean ± S.D. (n=5).


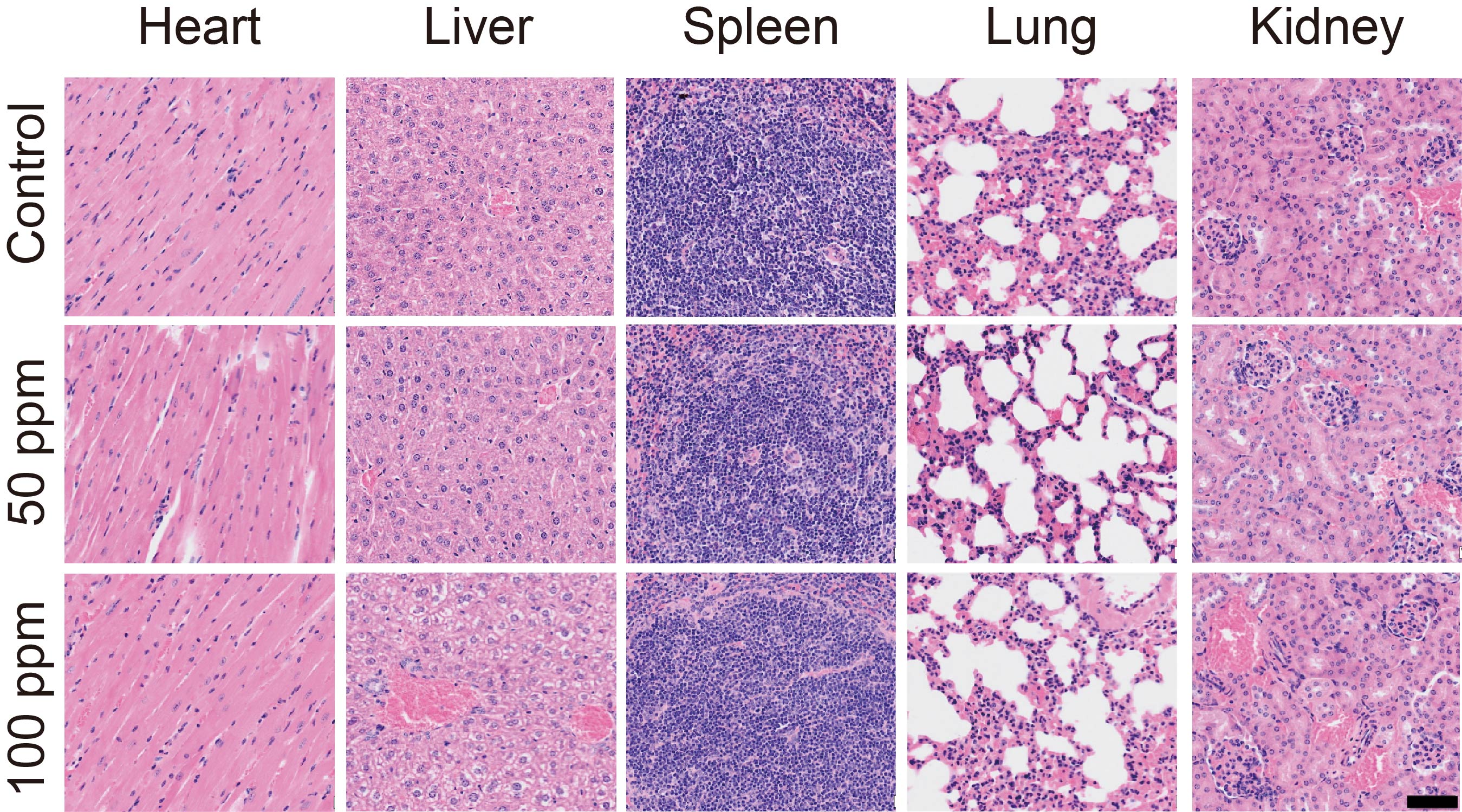


**Figure S27.** Representative H&E staining of heart, liver, spleen, lung, and kidney sections of mice in different groups. Scale bar: 50 μm.


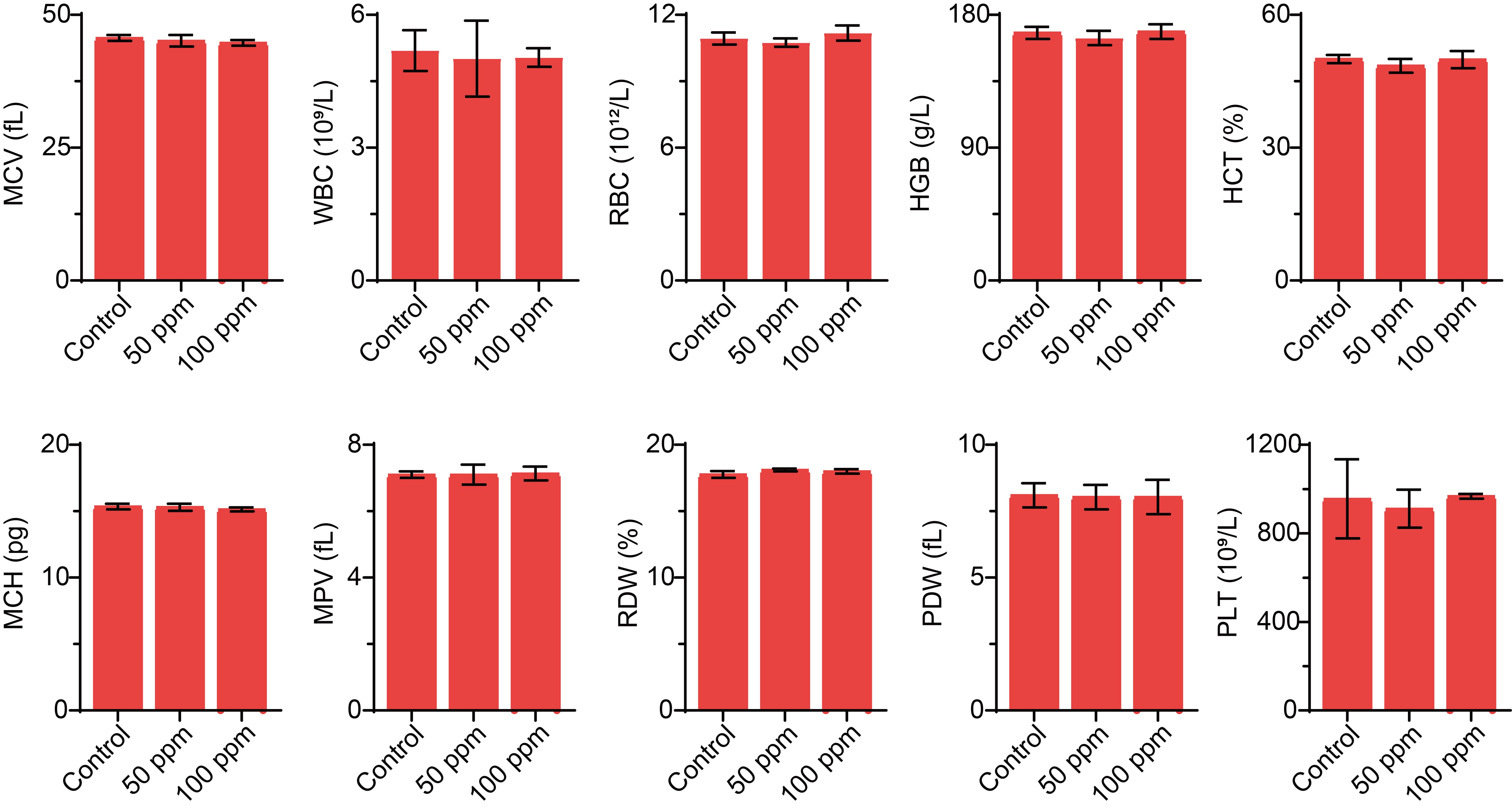


**Figure S28.** Blood routine examination of the mice in the different concentrations of GZn NPs groups. Data are presented as mean ± S.D. (n=3).

**
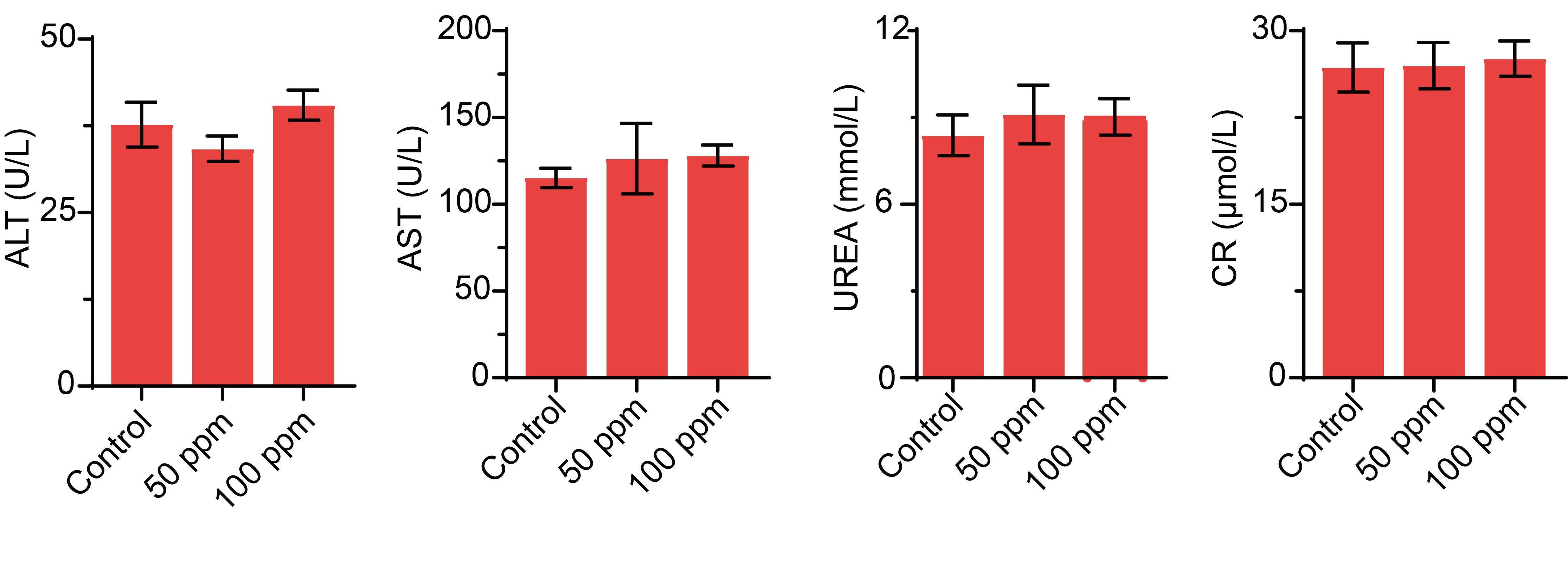
**

**Figure S29.** Blood biochemistry analyses of the mice in different groups. Data are presented as mean ± S.D. (n=3).


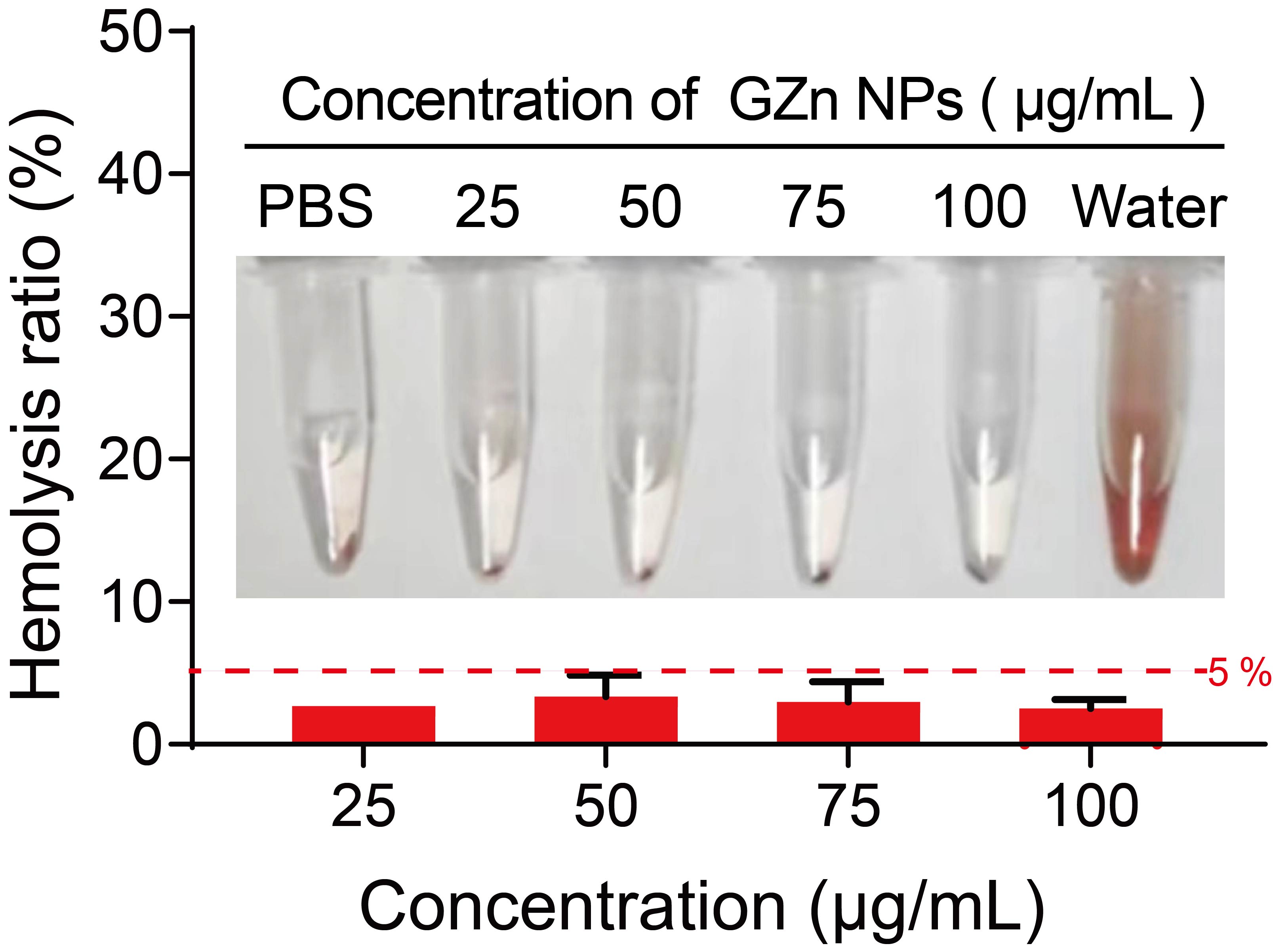


**Figure S30.** Hemolysis analysis of blood incubated with water (positive control), PBS (negative control), and the different concentrations of GZn NPs (inset: the corresponding digital photograph). Data are presented as mean ± S.D. (n=3).


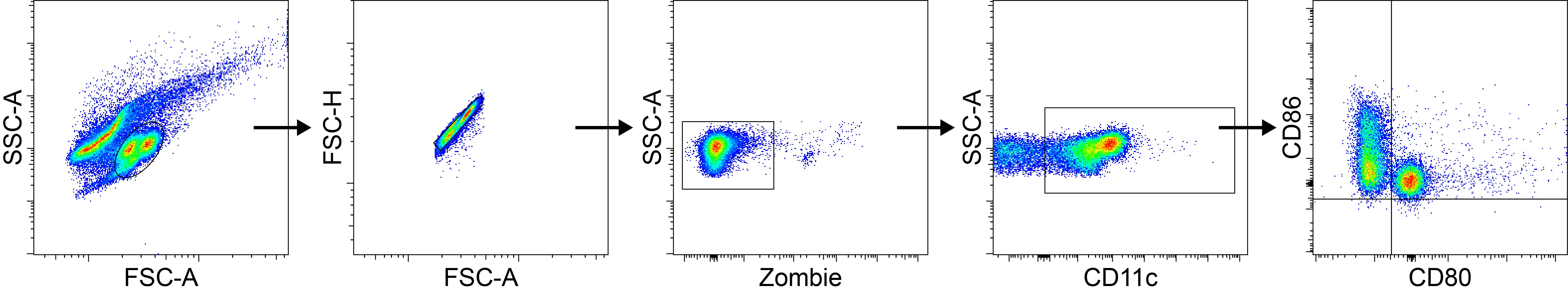


**Figure S31**. Gating strategy of CD11c^+^CD80^+^CD86^+^DC cells.


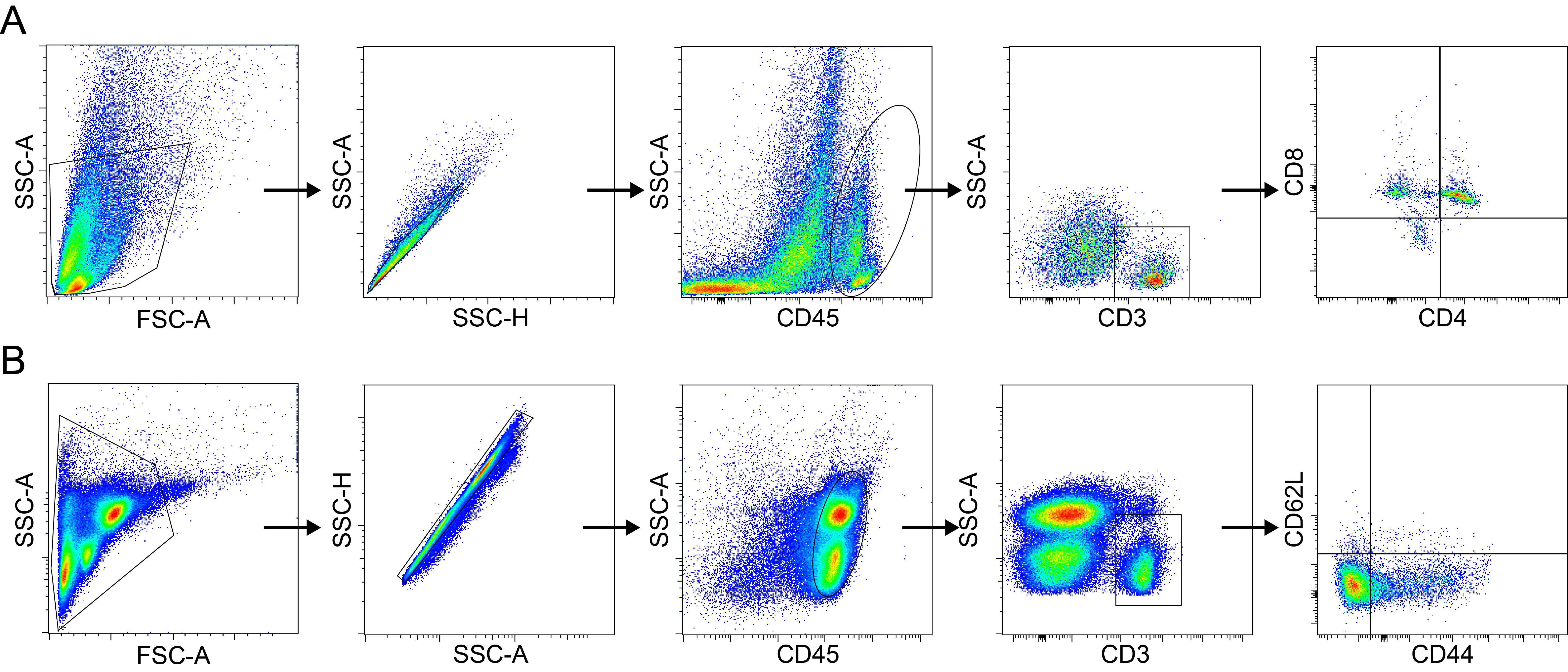


**Figure S32**. (A) Gating strategy to sort CD4^+^T cells and CD8^+^T cells. (B). Gating strategy for memory T cells.

**Supplementary Table 1. The primers used for quantitative real-time PCR analysis**

| Primer name | Sequence（5‘-3’） |
| --- | --- |
| mIFNB1-F | CAGCTCCAAGAAAGGACGAAC |
| mIFNB1-R | GGCAGTGTAACTCTTCTGCAT |
| mISG56-F | ACAGCAACCATGGGAGAGAATGCTG |
| mISG56-R | ACGTAGGCCAGGAGGTTGTGCAT |
| mTNFa-F | TAGCCCACGTCGTAGCAAAC |
| mTNFa-R | TGTCTTTGAGATCCATGCCGT |
| mlL-6-F | GGGACTGATGCTGGTGACAA |
| mIL-6-R | ACAGGTCTGTTGGGAGTGGT |
| mCxCL10-F | CCAAGTGCTGCCGTCATTTTC |
| mCxCL10-R | GGCTCGCAGGGATGATTTCAA |
| mActinb-F | TTCTTTGCAGCTCCTTCGTT |
| mActinb-R | ATGGAGGGGAATACAGCCC |
